# Structural basis of Ku-mediated activation of WRN exonuclease activity

**DOI:** 10.1038/s41467-026-71888-w

**Published:** 2026-05-13

**Authors:** Sayma Zahid, Jeanne Chauvat, Ilaria Ceppi, Floriana Cappiello, Benedetta Perdichizzi, Philippe Frit, Dennis Gomez, Steven W. Hardwick, Pierre Legrand, Julien Karazi, Sonia Baconnais, Gérard Pehau-Arnaudet, Sébastien Britton, Jean-Baptiste Charbonnier, Amanda K. Chaplin, Pietro Pichierri, Petr Cejka, Patrick Calsou, Virginie Ropars

**Affiliations:** 1 Université Paris-Saclay, CEA, CNRS, Institute for Integrative Biology of the Cell (I2BC), 91198 Gif-sur-Yvette, France; 2 Synchrotron SOLEIL, l’Orme des Merisiers, Saint Aubin, France; 3 Institut de Pharmacologie et Biologie Structurale, IPBS, Université de Toulouse, CNRS, UPS, Toulouse, France; 4 Institute for Research in Biomedicine, Università della Svizzera italiana (USI), Faculty of Biomedical Sciences, Bellinzona, Switzerland; 5 Mechanisms, Biomarkers and Models Section, Genome Stability Group, Department of Environment and Health, Istituto Superiore di Sanità, Viale Regina Elena 299, 00161 Rome, Italy; 6 Cryo-EM Facility, Department of Biochemistry, University of Cambridge, Sanger Building, Tennis Court Road, Cambridge, CB2 1GA UK; 7 Genome Integrity and Cancer UMR 9019 CNRS, Université Paris-Saclay, Gustave Roussy, 114 rue Edouard Vaillant, 94805 Villejuif, France; 8 UTECH UBI, Institut Pasteur and CNRS UMR, 3528 Paris, France; 9 Leicester Institute for Structural and Chemical Biology, Department of Molecular and Cell Biology, University of Leicester, Leicester, UK

**Keywords:** Stalled forks, Double-strand DNA breaks, Cryoelectron microscopy

## Abstract

Werner (WRN) is the only human RecQ helicase family member with DNA exonuclease activity. WRN promotes genome stability through its functions in DNA replication, repair and telomere maintenance, the deficiency of which presents clinically as Werner syndrome, causing premature aging and cancer predisposition. The main DNA double strand-break sensor Ku70/80 heterodimer (Ku) is a known partner of WRN, which stimulates its nuclease activity. However, the molecular basis of Ku-WRN interplay is currently unknown. Here, we present a high resolution cryo-EM structure of human Ku bound to DNA in complex with the N-terminal WRN exonuclease domain. This structure reveals multiple interaction sites between WRN and the Ku:DNA complex. The catalytic domain of WRN-exo engages with the DNA ends, stabilized by the vWA-like Ku80 domain interacting with the N-terminal APLF-like Ku binding motif (A-KBM) of WRN. Most surprisingly, we visualize the SAP domain of Ku70 stabilized within this complex, and we identify specific contacts mediating this interaction. These interactions are validated by assessing the impact of point mutations on either side of the Ku-WRN interfaces on exonuclease activity with purified recombinant proteins, and on live protein recruitment at biphoton laser-damaged nuclear sites. Finally, we show that disruption of WRN-Ku70 interaction results in aberrant resection of stalled replication forks. Together, we define the architecture of the Ku-WRN exonuclease domain interface and its impact on WRN exonuclease activity, recruitment and replication fork processing.

## Abstract (web summary)

The WRN helicase helps to protect stalled DNA replication forks during replication stress, but its activation mechanism remains unclear. Here, the authors demonstrate that Ku binds and stimulates WRN exonuclease activity, preventing fork degradation.

## Introduction

Werner (WRN) is a RecQ family protein with 3’ − 5’ helicase and 3’ − 5’ exonuclease activities that play roles in DNA metabolism during replication and repair^1–3^. WRN maintains telomere stability and also plays roles at stalled forks to counteract replication stress, thereby ensuring genomic stability (for reviews^3,4^). Mutations in WRN cause the autosomal-recessive disorder Werner syndrome characterized by premature age-associated diseases including cancer^5,6^. WRN function relies in part on its ability to disrupt non-canonical DNA structures including G-quadruplexes^7,8^. Recently, targeting WRN helicase activity has emerged as a potential strategy to eradicate tumors with unstable microsatellites that form cruciform structures, the replication of which strictly relies on WRN-mediated unfolding^9^.

In large-scale cellular interactome analyses, WRN appeared among the top candidates as partners of the DNA-end sensor Ku, a core protein of the non-homologous end-joining (NHEJ) pathway for the repair of DNA double-strand breaks (DSB)^10–13^. WRN has also been shown to interact with other NHEJ factors, including DNA-PKcs^14^ and XRCC4-DNA ligase IV complex^15^. The Ku interaction with WRN strongly stimulates WRN exonuclease activity in vitro^16–19^. DNA-PKcs phosphorylates WRN and regulates its enzymatic activities following binding to Ku and DNA ends^14,20^. Ku Binding Motifs (KBM) were identified at both the N- and C-termini of WRN via PSI-BLAST analysis^17^. Subsequently, it was shown that each of these WRN motifs in isolation is indeed able to bind the Ku heterodimer^21^. A recent X-ray structure of the N-terminal KBM of WRN in complex with the vWA domain of *Xenopus laevis* Ku80 (xlKu80)^22^ revealed that this WRN-KBM occupies the same site as the KBM of APLF (Aprataxin and PNKP-like factor) bound on human Ku heterodimer^23^.

Despite the presence of several KBMs in WRN and multiple reports documenting Ku-WRN interaction in cells, disruption of this interaction does not functionally translate into cell sensitivity to ionizing radiation, radiomimetic drugs, or topoisomerase II inhibitors^14,24–26^. Therefore, WRN plays a marginal, if any, role in NHEJ. It has been instead proposed that WRN prevents unfaithful alternative end-joining of DSBs by limiting MRN-dependent DNA resection at break ends^27^. Nevertheless, the precise function of Ku-WRN interaction in cells is unknown.

Ku plays multiple roles outside of NHEJ^28^. Among them, a function of Ku at perturbed replication forks is suggested by its presence at collapsed and reversed forks^29–37^ and by the sensitivity to replication stress observed upon Ku loss in various organisms^38–40^. The NHEJ-independent role of Ku at stressed forks may control resection and ensure proper repair by homologous recombination (reviewed in ref. ^29^). Notably, WRN is also enriched at stalled forks^41^ and its exonuclease and helicase activities^42–44^, as well as non-catalytic functions^45^ have been implicated in the resolution of collapsed or reversed replication forks. However, it is not yet known whether WRN function at stressed forks relies on its interaction with Ku.

In this study, we describe a high-resolution cryo-EM structure of human Ku bound to DNA end in complex with the N-terminal exonuclease domain of WRN. This structure highlights the interaction between Ku80 and the N-terminal APLF-like KBM of WRN, while also revealing specific contacts between WRN and the SAP domain of Ku70. To evaluate the functional significance of these interactions, we introduced point mutations at the Ku-WRN interfaces. We assessed the impact of these mutations on WRN exonuclease activity using purified proteins and on the recruitment of these proteins to biphoton laser-induced nuclear damage sites. Finally, we analyzed how these mutations influence resection at stressed replication forks. Our results decipher the structural basis of the Ku-driven activation of WRN exonuclease activity and argue for its role at stalled replication forks.

## Results

### Structure of the WRN N-terminal exonuclease domain bound to Ku-DNA

**Fig. 1:**
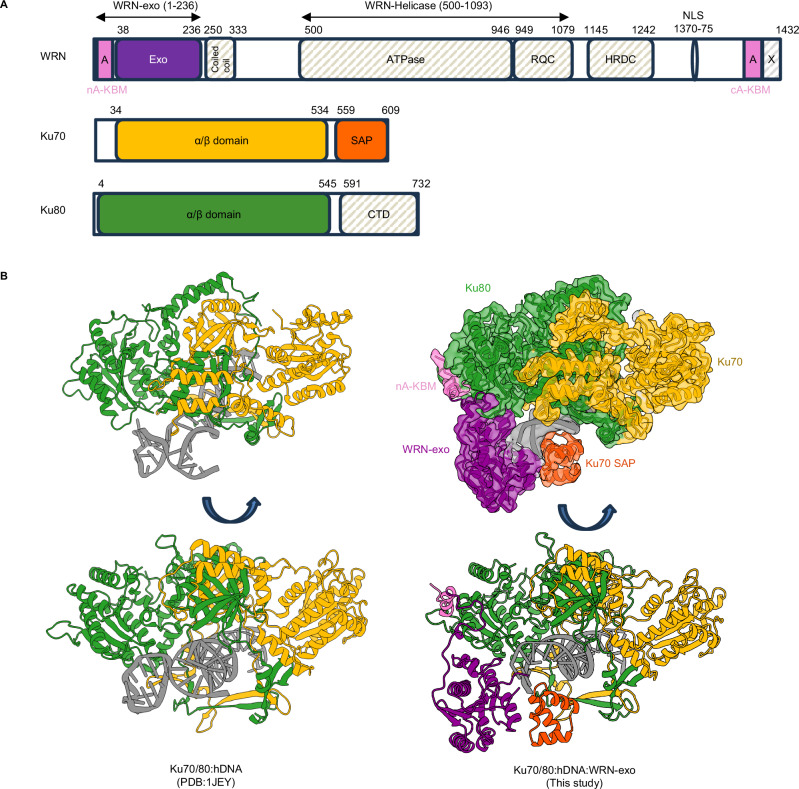
Cryo-EM structure of the Ku70/80-hDNA:WRN-exo complex. **A** Schematic representation of WRN, Ku70, and Ku80 sequence and constructs used in this study. Dashed gray domains are either deleted or not visible in the final structure. The C-terminal and N-terminal APLF-like Ku Binding Motifs are colored in pink. **B** Left, the X-ray crystal structure of Ku70/80 (yellow/green) bound to a hairpin DNA in gray (PDB 1JEY). Right, the cryo-EM structure of Ku70/80-DNA bound to WRN-exo (purple). The top panel shows the experimental map as a transparent surface. Structural domains are colored as in A.

To characterize the interaction between the Ku-DNA complex and the N-terminal region of WRN at the molecular level, we generated a human WRN construct, which encompasses its N-terminal Ku Binding Motif (nA-KBM) and the exonuclease domain, herein termed WRN-exo (Fig. 1A). We incubated WRN-exo with full-length human Ku70/80 bound to hairpin DNA (hDNA), which consists of a 14 bp duplex region and a 7 bp duplex region separated by a hairpin with a 4 bp stem and a 5 nt loop (Supplementary Fig. 1A^46^), and prepared cryo-EM grids of this complex sample. We collected data from one of these grids, and obtained a map with an overall resolution of 3.33 Å (Supplementary Fig. 1B–G). The map clearly shows that the Ku70/80 heterodimer engages DNA as previously described^46^, but additional density could be observed corresponding to WRN-exo and the SAP domain of Ku70 (Fig. 1B, Supplementary Fig. 5E). The nA-KBM of WRN can be seen bound to the outer surface of the vWA domain of Ku80, whilst the catalytic exonuclease domain of WRN is positioned on the end of the DNA chain away from the DNA end where a hairpin structure is expected. Finally, the SAP domain of Ku70 can be seen engaged with the DNA substrate and WRN-exo. The molecular details and functional consequences of these specific interactions are assessed in the following sections.

### Biophysical characterization of Ku interaction with WRN A-KBMs

**Fig. 2:**
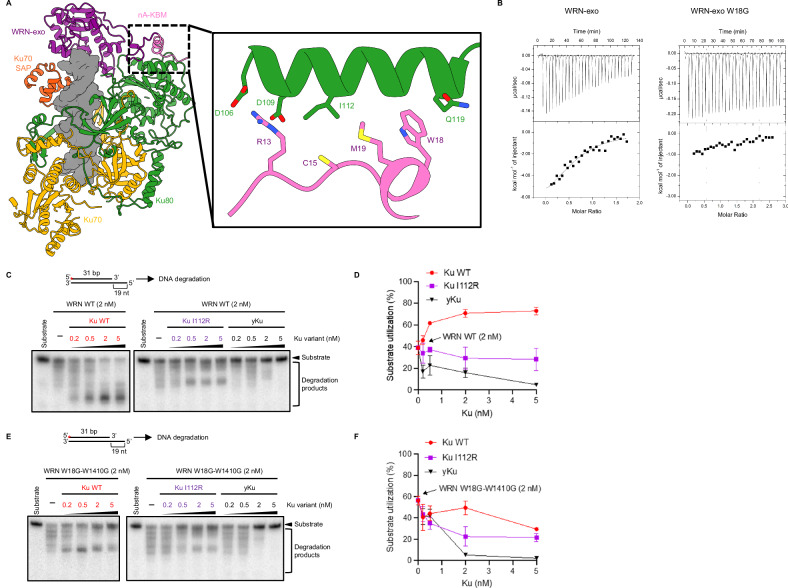
Role of WRN A-KBMs for interaction with Ku and for Ku-dependent stimulation of WRN exonuclease activity. **A** Overall structure of WRN-exo construct (purple) bound to Ku70/80 heterodimer (yellow/green) and DNA (gray). Zoomed in view of the interaction sites between the N-terminal KBM of the WRN-exo construct (nA-KBM in pink) and the vWA domain of Ku80 (green). **B** ITC measurements of KuFL-hDNA titrated by WRN-exo (left panel) and WRN-exo W18G (right panel). Top, thermogram. Bottom, enthalpies measurements versus molar ratio. **C** Top, schematic of the assay. Red asterisk indicates the position of the radioactive label. Bottom, representative 15% denaturing polyacrylamide gels showing the degradation of a 5ʹ overhang (19 nt/31 bp) DNA by WRN WT in the presence of human Ku WT or I112R mutant, or *S. cerevisiae* yeast Ku (yKu) as indicated. **D** Quantification of overall substrate degradation from experiments such as shown in C. Averages shown; *n* = 3 independent experiments; error bars, SEM. **E** Top, schematic of the assay. Red asterisk indicates the position of the radioactive label. Bottom, representative 15% denaturing polyacrylamide gels showing the degradation of a 5ʹ overhang (19 nt/31 bp) DNA by WRN W18G-W1410G mutant in the presence of human Ku WT or I112R, or yeast Ku (yKu) as indicated. **F** Quantification of overall substrate degradation from experiments such as shown in E. Averages shown; *n* = 3 independent experiments; error bars, SEM. B-F: Source data are provided as a Source Data file.

Our cryo-EM structure of Ku70/80-DNA in complex with WRN-exo clearly shows the nA-KBM binding to the periphery of the vWA domain of Ku80 (Fig. 2A, Supplementary Fig. 5E), consistent with the crystal structure of the vWA domain of xlKu80 engaged with the WRN nA-KBM^22^. The WRN nA-KBM occupies a hydrophobic groove formed between α4 and α5 of the vWA domain of Ku80, with isoleucine 112 of Ku80 packing against cysteine 15 and methionine 19 of WRN nA-KBM. Other prominent residues at this interaction interface are arginine 13 of WRN nA-KBM packing against aspartates 106 and 109 of Ku80, and the conserved tryptophan 18 of WRN nA-KBM in close proximity to glutamine 119 of Ku80 (Fig. 2A, Supplementary Fig. 2A).

To assess the importance of the nA-KBM in the interaction between WRN and Ku70/80, relative to other KBMs of WRN, we used isothermal calorimetry (ITC) with full-length Ku70/80 (KuFL) protein and various WRN N-terminal (n) and C-terminal (c) peptides which contained APLF (A)- and/or XLF (X)-like KBMs (WRN nA, WRN cA, WRN cX, and WRN cAX, respectively) (Supplementary Fig. 2B). WRN nA and cA peptides bound to KuFL with micromolar affinity: 2.8 ± 0.6 µM and 0.5 ± 0.1 µM, respectively (Supplementary Fig. 2C, D). Indeed, AlphaFold3^47^ predicted a model for cA interaction on Ku80 very similar to the structure we obtained for nA (Supplementary Fig. 2E-G). No interaction was observed between KuFL and WRN cX peptide, predicted to be a XLF-like motif that would bind to the inner face of the Ku80 vWA domain^23^. In addition, WRN cAX peptide exhibits micromolar affinity for KuFL, similar to that measured for WRN cA alone (Supplementary Fig. 2C-D). Thus, the interaction of WRN cAX with Ku in vitro is primarily driven by the A-KBM motif. The lack of cooperativity between WRN cX- and cA-KBMs found by ITC is in agreement with previous fluorescence polarization data^17^.

We then performed ITC measurements with the N-terminal region of WRN in isolation (WRN-exo) and the KuFL in complex with hDNA at a 1:1.2 ratio. WRN-exo had an affinity for KuFL (Kd = 2.0 ± 0.55 µM) similar to that of WRN nA (Fig. 2B and Supplementary Fig. 2D). When the conserved tryptophan in WRN nA (WRN W18G) was mutated in the WRN-exo construct, no detectable dissociation constant was measured, indicating loss of binding affinity (Fig. 2B and Supplementary Fig. 2D).

### Interaction between Ku80 vWA domain and WRN A-KBMs stimulates WRN exonuclease in vitro

To assess the functionality of the KBM-dependent WRN-Ku interaction, we set up an exonuclease assay with purified proteins (Supplementary Fig. 3A) and radiolabeled DNA substrates (Supplementary Fig. 3B–F), similar to those described previously^16^. Under our conditions, full-length human WRN protein exhibited a concentration-dependent 3’−5’ exonuclease activity, both on a DNA substrate carrying a single-stranded overhang at the 5’ end (31 bp + 19 nt 5’ overhang) and on a blunt ended DNA substrate (50 bp) (Supplementary Fig. 3B, D–F). This activity relied on the integrity of the WRN exonuclease domain since it was abolished by the E84A mutation in the catalytic pocket (Supplementary Fig. 3B, C). Addition of human full-length Ku promoted the WRN exonuclease activity on the 5’-overhang substrate (Fig. 2C, D and Supplementary Fig. 3D), while addition of yeast Ku was strongly inhibitory (Fig. 2C, D and Supplementary Fig. 3E), supporting the notion that species-specific interaction underlies the observed stimulatory effect. Human and yeast Ku exhibited similar DNA binding activities as assessed by electromobility shift assay (Supplementary Fig. 3G). In contrast to the stimulation of WRN on the 5’-overhang substrate, human Ku inhibited WRN exonuclease-dependent degradation of the 50 bp-long double blunt DNA (Supplementary Fig. 3F).

Ku binding to DNA ends is oriented with Ku70 outward and Ku80 inward from the break end^48^. When superimposed onto models of DNA-PK, our structural data indicate that the interaction between WRN-exo and Ku70/80 positions the exonuclease domain on the internal face of Ku70/80 with respect to the DNA break end; i.e. on the other side than DNA-PK (Fig. 1B and Supplementary Fig. 5A). Thus, we hypothesized that the stimulation of WRN exonuclease activity on the 5’-overhang substrate in the presence of Ku70/80 is most likely due to the preferential binding of one Ku dimer at the blunt end of the DNA substrate, subsequently positioning WRN-exo to attack the 3’ end of the shorter DNA strand (top strand in Fig. 2C). Indeed, when the blunt end of the 5’- protruding substrate was blocked with streptavidin, Ku70/80 was no longer able to stimulate the exonuclease activity of WRN, likely because Ku70/80 was not able to bind the blunt end (Supplementary Fig. 4A, compare middle panel with side panels). Furthermore, when we extended the length of the central duplex DNA region, the stimulatory effect was diminished (Supplementary Fig. 4A), arguing that DNA end-bound-Ku might need to physically engage WRN bound to the overhang.

Next, we questioned whether the KBM-mediated direct interaction between WRN and Ku70/80 was indeed necessary for the Ku-dependent stimulation of WRN exonuclease activity. Guided by our cryo-EM structural data (Fig. 2A), we generated mutations at the interface between WRN and Ku. Specifically, isoleucine 112 of the Ku80 vWA domain was mutated to arginine, a position previously reported to be essential for the interaction with the KBM of APLF^23,49^. While the Ku I112R mutant protein bound DNA ends as efficiently as Ku WT (Supplementary Fig. 3G), it was unable to stimulate the exonuclease activity of WRN (Fig. 2C, D). Conversely, we disrupted both WRN A-KBMs by mutating the conserved tryptophan in WRN nA and WRN cA to glycine (Supplementary Fig. 3A). While WRN W18G-W1410G double mutant in isolation exhibited exonuclease activity comparable to the WT protein (Supplementary Fig. 3B, C), it was not stimulated by either human Ku, WT, or I112R (Fig. 2E, F and Supplementary Fig. 4B).

### WRN N-terminal A-KBM dictates the Ku-dependent recruitment of WRN-exo domain to DNA damage sites in cells

**Fig. 3:**
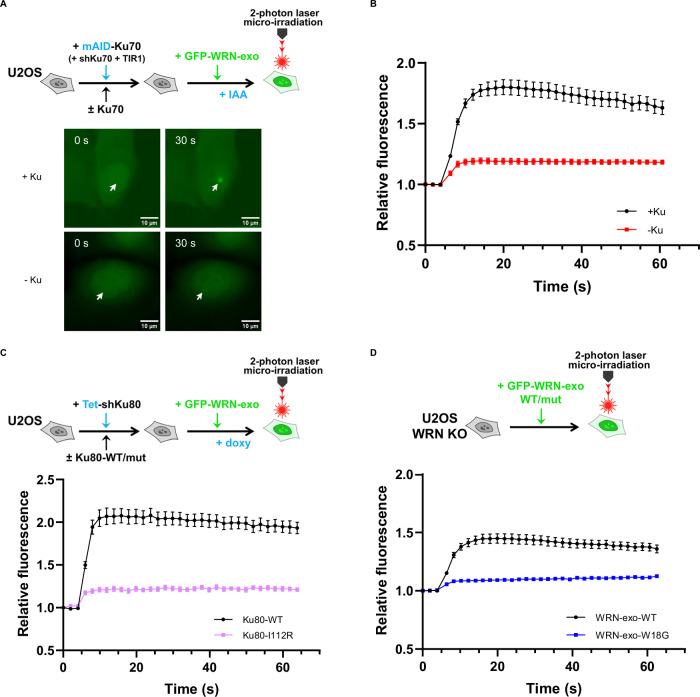
Role of WRN A-KBMs for Ku-dependent recruitment to DNA damage sites. **A** Top, principle of the laser micro-irradiation experiment. U2OS cells engineered for auxin (IAA)-induced Ku70 knockdown and expressing either GFP-tagged WT or mutant WRN-exo, were micro-irradiated with an 800 nm multiphoton laser to generate DSBs in subnuclear areas. Bottom, representative images before and 30 s after irradiation (irradiated areas are indicated by white arrowheads) of nuclei from U2OS expressing GFP-tagged WRN-exo and depleted or not of Ku70 ( ± Ku). Scale bar: 10 μm. **B** Quantification of WT WRN-exo recruitment (fluorescence accumulation) at laser-induced DNA damage sites under Ku+ or Ku- conditions, as indicated. Results are plotted as mean values of 25 nuclei ( + Ku) and 47 nuclei (-Ku) ± SEM. **C** Top, principle of the laser micro-irradiation experiment. U2OS cells expressing an inducible shRNA against Ku80 responsive to doxycyclin addition, sh-resistant WT or mutant Ku80 and GFP-WRN-exo were micro-irradiated and accumulation of fluorescence was analyzed. Bottom, quantification of WT WRN-exo recruitment (fluorescence accumulation) at laser-induced DNA damage sites. Results are plotted as mean values of 20 nuclei (WT Ku80) and 18 nuclei (I112R mutant) ± SEM. **D** Top, principle of the laser micro-irradiation experiment. U2OS cells knocked out for WRN and expressing WT or mutant GFP-tagged WRN-exo were micro-irradiated, and the accumulation of fluorescence was analyzed. Bottom, quantification of WT and mutant WRN-exo recruitment (fluorescence accumulation) at laser-induced DNA damage sites. Results are plotted as mean values of 23 nuclei (WT WRN-exo) and 20 nuclei (W18G mutant) ± SEM. A-D: Source data are provided as a Source Data file.

Based on our structural data, we next assessed the impact of the A-KBM-mediated WRN interaction with Ku70/80 in cells. We analyzed the recruitment of the WRN-exo fragment fused to GFP (GFP-WRN-exo) at laser-induced DNA damage sites. To determine the extent to which WRN-exo mobilization to damaged sites was Ku-dependent, we used in-house-engineered U2OS cells in which the essential endogenous Ku70 was replaced with Ku70 tagged with a mini-auxin inducible degron (mAID). Within a few hours upon addition of indole-3-acetic acid (auxin, IAA), degradation of mAID-Ku70 occurs, accompanied by destabilization of Ku80^50^. Since the rapid recruitment of GFP-WRN-exo to micro-irradiated areas was nearly abolished without Ku, we concluded that it was mainly Ku-dependent (Fig. 3A, B). Based on our structural and biochemical data, the impact of specific mutations at the Ku:WRN-exo interface on Ku-dependent WRN-exo recruitment to DNA damage sites was monitored (Fig. 3C, D). Either the Ku80 I112R mutation in the nA-KBM binding site or the W18G mutation in the WRN nA-KBM strongly compromised WRN-exo recruitment to nuclear DNA damage sites (Fig. 3C, D). Importantly, recruitment to damaged DNA sites of Ku itself, as measured by recruitment of mCherry-Ku70, was not compromised by the Ku80 I112R mutation, showing that the impairment of WRN-exo recruitment was primarily due to a disruption of the interaction between Ku and WRN (Supplementary Fig. 4C). Altogether, these data indicate a major role of the A-KBM-mediated WRN interaction with Ku for WRN mobilization to damaged DNA and subsequent stimulation of WRN exonuclease activity.

### Ku-dependent stimulation of WRN exonuclease occurs during cryo-EM sample preparation

A hairpin DNA substrate was used for the preparation of our cryo-EM samples (Supplementary Fig. 1A). We modeled the hairpin region close to the vWA domain of Ku80 based on the crystal structure obtained for Ku-hDNA-pXLF, which used the same hairpin substrate (PDB: 6ERG^23^,) (Supplementary Fig. 5B). The Ku70 subunit is positioned near the blunt DNA end, while the Ku80 subunit is located near the hairpin. In the Ku-hDNA:WRN-exo complex, only the density of the 14 bp duplex was clearly visible (colored in red in Supplementary Fig. 5C). Unlike the Ku-hDNA-pXLF structure, we were unable to resolve the hairpin region of the DNA molecule. In the 3’ DNA end closest to the hairpin, three nucleotides were missing, and this end was positioned approximately 15 Å away from the active site of the WRN exonuclease domain (Fig. 1B, Supplementary Fig. 5E). We reasoned that the hairpin may have been resected during the mixing phase of purified proteins and DNA prior to cryo-EM sample vitrification. Therefore, we performed the WRN exonuclease assay with WRN-exo alone or in the presence of Ku on the hairpin DNA (Supplementary Fig. 5C, D). Indeed, Ku stimulated WRN exonuclease activity from the 3’ to the 5’ end of hDNA, most likely explaining the lack of hairpin detection in the structure we obtained (Supplementary Fig. 5D, E).

### The Ku70 SAP domain stimulates the exonuclease activity of WRN in vitro

**Fig. 4:**
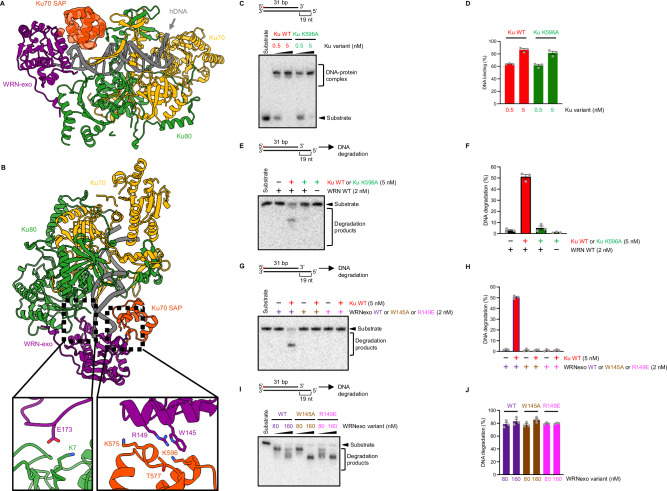
Ku70 SAP domain contributes to stimulation of WRN exonuclease by Ku. **A** View of the interaction between WRN-exo (purple) and the SAP domain of Ku70 (orange) with Ku70/80 core region. The models are fitted in the cryo-EM map. **B** Zoom of the interaction between WRN-exo (purple), Ku80 (green) and the SAP domain of Ku70 (orange). The amino acids outlined correspond to the mutated positions in the following experiments. **C** Top, schematic of the DNA substrate. Red asterisk indicates the position of the radioactive label. Bottom, representative 4% native polyacrylamide gel showing the electrophoretic mobility shift of a 5ʹ overhang (19 nt/31 bp) DNA substrate by human Ku WT or K596A mutant. **D** Quantification of DNA binding from experiments such as shown in C. Averages shown; *n* = 3 independent experiments; error bars, SEM. **E** Top, schematic of the assay. Red asterisk indicates the position of the radioactive label. Bottom, representative 15% denaturing polyacrylamide gel showing the degradation of a 5ʹ overhang (19 nt/31 bp) DNA by WRN WT in the presence of human Ku WT or K596A mutant. **F** Quantification of DNA degradation from experiments such as shown in E. Averages shown; *n* = 3 independent experiments; error bars, SEM. **G** Top, schematic of the assay. Red asterisk indicates the position of the radioactive label. Bottom, representative 15% denaturing polyacrylamide gel showing the degradation of a 5ʹ overhang (19 nt/31 bp) DNA by WRN-exo WT or W145A or R149E mutant in the presence of human Ku WT. **H** Quantification of DNA degradation from experiments such as shown in G. Averages shown; *n* = 3 independent experiments; error bars, SEM. **I** Top, schematic of the assay. Red asterisk indicates the position of the radioactive label. Bottom, representative 15% denaturing polyacrylamide gel showing the degradation of a 5ʹ overhang (19 nt/31 bp) DNA by WRN-exo WT or W145A or R149E mutant. **J** Quantification of DNA degradation from experiments such as shown in I. Average shown; *n* = 3 independent experiments; error bars, SEM. C-J: Source data are provided as a Source Data file.

A surprising observation from our cryo-EM structure of the Ku-hDNA:WRN-exo complex was that the SAP domain of Ku70 can be visualized and is in contact with both the DNA substrate and the WRN exonuclease domain (Fig. 1B and Supplementary Fig. 5E). Amino acid W145 of WRN docks against T577 and K596 of the SAP domain (Fig. 4A, B and Supplementary Fig. 6A). The residues K596 and T577 of Ku70 involved in these interactions are well conserved (Supplementary Fig. 6A). Notably, although the SAP domain of Ku70 was positioned near WRN exonuclease domain by AlphaFold3 as observed in our cryo-EM structure, the predicted SAP domain orientation was different, with no contact between the residue K596 of Ku70 and WRN exonuclease domain (Supplementary Fig. 6B).

To assess the functional relevance of the Ku70 SAP domain on WRN function, the exonuclease activity of WRN was first measured in the presence of Ku with both Ku70 and Ku80 C-terminal domains deleted (Ku CC) (Supplementary Fig. 6C). We have previously shown that Ku CC is not impaired in DNA binding activity (Supplementary Fig. 6D)^23^. Unlike with full-length Ku70/80, the addition of the Ku CC construct resulted in no stimulation of WT WRN exonuclease activity, and even a strong inhibition with the A-KBM mutated version of WRN (W18G-W1410G) was observed (Supplementary Fig. 6E–H). Next, to rule out any possible impact of the deletion of the Ku80 C-terminal domain, we mutated KuFL at various Ku and WRN residues deduced from the structure of the Ku70 SAP-WRN interface (Fig. 4B). Although Ku K596A mutant (SAP domain) exhibited DNA binding activity identical to WT Ku (Fig. 4C, D), it no longer stimulated WRN exonuclease activity (Fig. 4E, F). Similarly, W145A and R149E WRN-exo mutant proteins were no longer stimulated by WT Ku (Fig. 4G, H), while they showed identical substrate degradation activity without Ku (Fig. 4I, J). These data show that the interaction of the Ku70 SAP domain with WRN contributes to the stimulation of WRN exonuclease activity in vitro.

### The Ku70 SAP domain contributes to WRN recruitment to DNA damage sites

**Fig. 5:**
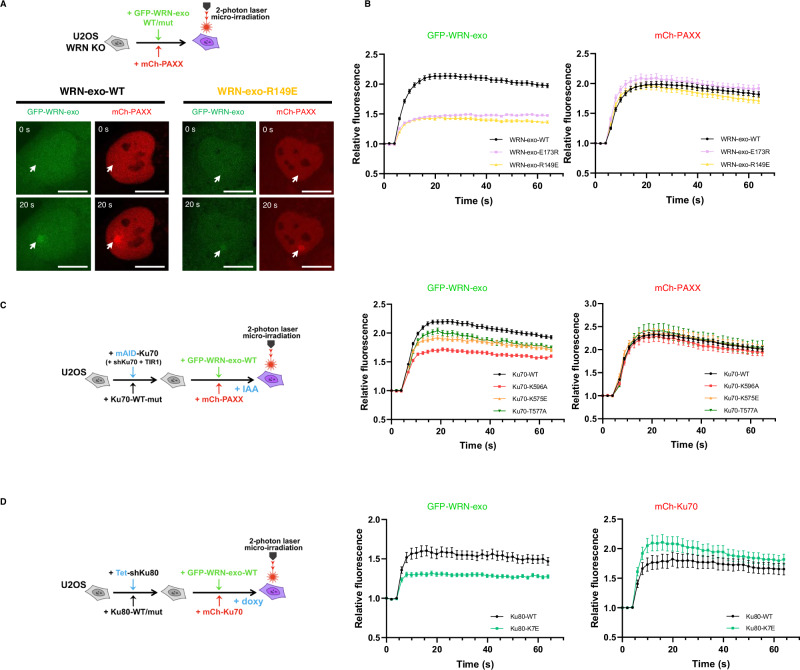
Ku70 SAP domain contributes to WRN recruitment to DNA damage sites. **A** Top, principle of the laser micro-irradiation experiment. U2OS cells knocked-out for WRN and coexpressing WT or mutant GFP-tagged WRN-exo and mCherry-tagged PAXX, were micro-irradiated and accumulation of each fluorescence was analyzed. Bottom, representative images before (upper frames) and 20 s after irradiation (lower frames, a white arrow indicates irradiated area) of nuclei from U2OS WRN KO expressing WT or mutant GFP-WRN-exo. Scale bar: 10 μm. **B** Quantification of fluorescence accumulation at laser-induced DNA damage sites of GFP-WRN-exo (left chart) and mCherry-PAXX (right chart) in U2OS WRN KO cells. Results are plotted as mean values of 74 nuclei (WT WRN-exo), 53 nuclei (E173R WRN-exo mutant) and 52 nuclei (R149E WRN-exo mutant) ± SEM. **C** Left, principle of the laser micro-irradiation experiment. U2OS cells engineered for auxin (IAA)-induced Ku70 knockdown, rescued with WT or mutant Ku70 and co-expressing GFP-WRN-exo and mCherry-tagged PAXX, were micro-irradiated and accumulation of each fluorescence was analyzed. Right, results are plotted as mean values of 130 nuclei (WT Ku70), 45 nuclei (K596A Ku70 mutant), 43 nuclei (K575E Ku70 mutant) and 44 nuclei (T577A Ku70 mutant) ± SEM. **D** Left, principle of the laser micro-irradiation experiment. U2OS cells engineered for Ku80 knockdown upon doxycyclin addition (doxy), rescued with WT or mutant Ku80 and co-expressing GFP-WRN-exo and mCherry-Ku70, were micro-irradiated and accumulation of each fluorescence was analyzed. Right, results are plotted as mean values of 16 nuclei (WT Ku80) and 31 nuclei (K7E Ku80 mutant) ± SEM. A-D: Source data are provided as a Source Data file.

To validate the key residues identified at the WRN-exo:Ku interface outside the A-KBM in a cellular context, we used the GFP-WRN-exo recruitment assay at laser-damaged nuclear areas in U2OS cells. We then assessed the impact of mutations in either WRN-exo (Fig. 5A, B) or Ku subunits (Fig. 5C, D, Supplementary Fig. 7D), as guided by our structural data. To ensure the integrity of the NHEJ complex at damaged sites in the presence of Ku mutants, we simultaneously monitored the recruitment of mCherry-PAXX, a Ku partner that was co-expressed within the same cells (Fig. 5A–C) (PAXX recruitment at laser-induced DNA damage is strictly Ku-dependent^51^). Regarding the mutations in Ku80, we monitored the recruitment of mCherry-Ku70 as a control (Fig. 5D). While PAXX recruitment was preserved, WRN-exo recruitment was significantly impaired by the E173R and R149E mutations in WRN-exo as well as by mutations in Ku70 SAP domain, with K596A being the most detrimental while supporting normal PAXX recruitment (Fig. 5B–D, Supplementary Fig. 7A and C). In addition, the K7E mutation in Ku80 impaired GFP-WRN-exo recruitment while preserving Ku recruitment to laser-damaged sites (Fig. 5D and Supplementary Fig. 7C). However, the impact of these mutations was less severe than that of the W18G mutation in WRN-exo or the I112R mutation in Ku80, both of which compromising the binding of WRN N-terminal A-KBM to Ku (Fig. 3C, D). It should be noted that the K596E and Q597A mutations in Ku70 SAP domain impaired PAXX recruitment (Supplementary Fig. 7B). Since residues 590-604 in SAP domain are also involved in DNA interaction^52,53^, this impairment may reflect a disruption of the interaction between Ku and DNA.

### Disruption of Ku-WRN interaction impacts resection at stressed replication forks

**Fig. 6:**
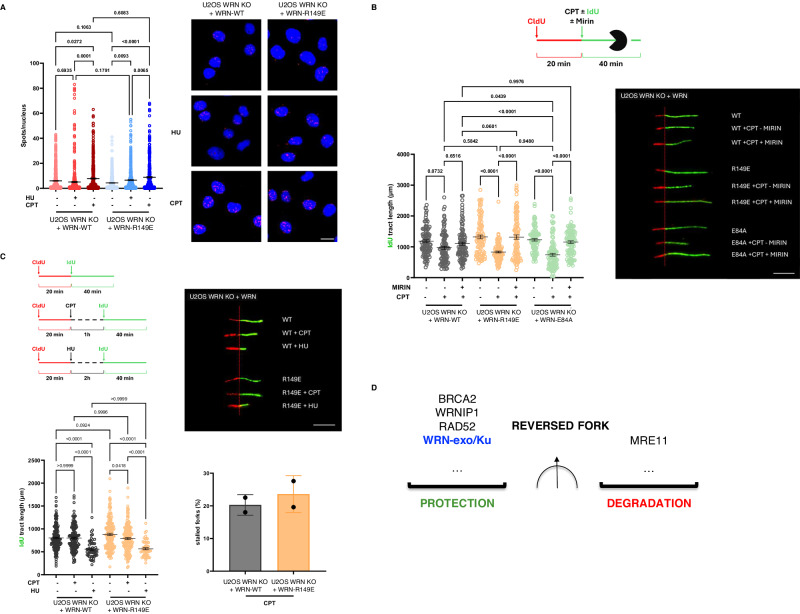
Impact of Ku-WRN interaction on resection at stalled replication forks. **A** SIRF PLA analysis of WRN localization at EdU-labeled DNA in U2OS WRN KO cells expressing wild-type or R149E WRN protein. PLA spots derive from at least 450 cells (three independent experiments). Bars represent mean ± S.E.M. Representative images on the right (scale bar: 20 μm). **B** Schematic of DNA fibers assay and IdU tract length analysis in U2OS WRN KO cells expressing wild-type, R149E or E84A WRN protein to assess fork degradation. Dot plots show IdU tract lengths (μm) from three independent replicates. Bars represent mean ± S.E.M. Representative fibers images on the right (scale bar: 10 μm). **C** Top left, schematic of DNA fibers assay and IdU tract length analysis in U2OS WRN KO cells expressing wild-type or R149E WRN protein to assess fork recovery. Top right, representative fibers images (scale bar: 10 μm). Bottom left, dot plots display IdU tract lengths (μm) from three independent replicates. Bars represent mean ± S.E.M. Bottom right, the percentage value of stalled forks was calculated by measuring the ratio between the number of CldU-labeled fibers and the number of complete fibers (CldU- and IdU-labeled fibers) x 100 from two independent replicates (central panel). A-C: Source data are provided as a Source Data file. Significance was assessed using the built-in tools in Prism 10 (GraphPad Inc.) by one-way ANOVA followed by Tukey’s two-sided multiple comparisons test. *P* < 0.05 was considered significant. Exact P values for the comparisons indicated in the graphs are reported above each comparison. P values were automatically adjusted using Prism 10. **D** Scheme of the balance between protection and degradation at a reversed fork, including the present report of the Ku-mediated regulation of WRN exonuclease activity.

Since localization of WRN to laser-induced DNA damage depends on a functional Ku70 interaction domain, and since the exonucleolytic activity of WRN has been shown to protect stalled replication forks from degradation under low dose of campthotecin (CPT)^43^, we investigated whether WRN-Ku70 interaction was also involved in the WRN recruitment at CPT-induced stalled replication forks. To this end, U2OS WRN KO cells were complemented with plasmids expressing WRN wild type or the R149E mutant (Supplementary Fig. 8A) and were subsequently analyzed for the recruitment at the replication fork by the SIRF (in situ Protein Interaction with Nascent DNA Replication Forks) assay (Fig. 6A, see left of Supplementary Fig. 8C for a scheme depicting the method). As expected, WRN was found already localized at DNA replication forks in untreated cells (Fig. 6A). Treatment with CPT resulted in enhanced WRN localization at stalled replication forks (Fig. 6A). Despite the impaired binding to Ku70, the WRN-R149E mutant was fully proficient in binding to DNA replication forks either in untreated conditions or after treatment with CPT (Fig. 6A). Similarly, localization at HU-stalled forks was not affected by the mutation preventing interaction with Ku70.

Given that WRN-Ku interaction stimulates exonuclease activity, which is required to protect reversed forks from being pathologically degraded^43^, we used native nascent ssDNA-PLA to monitor the association of WRN with ssDNA exposed at the regressed arm of the reversed fork in cells expressing the Ku70 SAP domain mutant K596A. While SIRF detects proteins associated to nascent DNA at replication fork, native nascent ssDNA PLA can detect protein associated only with exposed ssDNA in the nascent strand as it forms at reversed forks (see scheme on right of Supplementary Fig. 8C). In these cells, endogenous Ku70 was tagged with the mAID degron and, after supplementation with IAA, only the ectopic untagged Ku70 (either WT or mutant) was expressed (Supplementary Fig. 8B). Since the regressed arm of the reversed forks is composed of the two reannealed nascent DNA strands, degradation unveils nascent ssDNA labeled with IdU (see model on right of Supplementary Fig. 8C). Thus, nascent ssDNA (i.e. anti-IdU-WRN) PLA specifically identifies the fraction of WRN recruited at reversed forks to protect them from aberrant degradation. As shown in Supplementary Fig. 8C, WRN was significantly more localized at sites of nascent ssDNA after treatment with 50 nM CPT. Expression of the K596A Ku70 protein resulted in only a minor reduction in the localization of WRN at nascent ssDNA as seen by the number of PLA spots after CPT treatment (Supplementary Fig. 8C).

To indirectly estimate if reversed forks were not reduced in Ku70-K596A mutant, we performed nascent ssDNA PLA with anti-RAD51 antibodies. While RAD51 can be found at parental ssDNA at the fork or behind it, it can be detected at nascent ssDNA in reversed forks. PLA evaluating nascent ssDNA-RAD51 interaction was stimulated by CPT treatment and even more in cells expressing the Ku70 SAP mutant K596A (Supplementary Fig. 8D). Consistent with the SIRF data obtained in cells expressing the R149E WRN mutant, abrogation of the association with Ku70 did not affect WRN localization at stalled forks (Supplementary Fig. 8E). Expression of the exo-dead E84A WRN mutant results in fork degradation even if its interaction with nascent ssDNA is unaffected^43^. Thus, we analyzed whether the expression of the R149E WRN mutant was sufficient to phenocopy the fork degradation phenotype associated with the exo-dead WRN. DNA fibers were prepared from U2OS WRN KO cells expressing the wild type or the R149E mutant WRN protein after sequential labeling of ongoing forks with CldU and IdU (see scheme in Fig. 6B). As shown in Fig. 6B, quantification of the IdU tract length, which is shorter under degradation, indicated that treatment with 50 nM CPT significantly reduced the length of the IdU tract when the interaction between WRN and Ku70 was perturbed by the R149E mutation (Fig. 6B). These data clearly indicate that WRN R149E cannot protect nascent DNA from degradation, likely because its exonuclease activity fails to be stimulated by WRN.

The IdU tract length in WRN R149E cells was fully recovered by treatment with the MRE11 inhibitor MIRIN, indicating that MRE11 contributes to the nascent DNA degradation. As expected, the presence of the E84A mutant greatly stimulated fork degradation and the level of reduction in the IdU tract length in CPT-treated cells was similar between the two WRN variants (Fig. 6B). We next analyzed if abrogating the WRN-Ku70 interaction also affected the ability of CPT-perturbed replication forks to restart DNA synthesis by the DNA fiber assay. Since the seminal work of Jasin’s lab, it is known that the fork degradation phenotype is uncoupled from the ability to restart replication once the drug that induces fork stalling is washed out because of alternative mechanisms of fork restart^54,55^. The U2OS WRN KO cells complemented with the wild type or the R149E mutant WRN protein were labeled as shown in the scheme of Fig. 6C and fork recovery was evaluated by quantification of the IdU tract-length observed in dual-labeled replication tracks, and by quantification of the number of stalled forks represented by replication tracks with only the CldU tract. As shown in Fig. 6C, IdU tracts were of similar length in restarting forks analyzed from cells expressing the wild type or R149E WRN after CPT treatment. As a control, cells were also treated with 2 mM HU for 2 h, without any significant difference in the length of restarting forks between the wild-type or the WRN-R149E. Consistent with this quantification, also the percentage of stalled forks did not differ between the two genotypes.

Altogether, these results show that abrogation of the WRN-Ku70 interaction does not affect the ability of WRN to be recruited at stalled forks or, more specifically, at the nascent ssDNA formed at reversed forks. However, the interaction with Ku70 is essential for WRN to protect reversed forks from being pathologically degraded by MRE11.

## Discussion

The Werner protein contains several KBMs that directly interact with Ku^17^ including two APLF-like KBMs in the N-terminal and the C-terminal regions (nA and cA-KBMs), as well as a XLF-like KBM positioned in tandem with the cA-KBM (cX-KBM) (Fig. 1A). We report here a structure of the interface between the N-terminal region of human WRN, including the nA-KBM and its exonuclease domain, and Ku70/80. Notably, we found that the WRN nA-KBM interacts with the vWA domain of Ku80 via both backbone and side-chain contacts, providing insight into the molecular basis of this interaction. Our data include human full-length Ku and WRN-exo domain, which extends a previous report with the isolated xlKu80 vWA domain complexed with a hWRN nA-KBM peptide^22^. Furthermore, our cryo-EM structure reveals the detailed molecular interaction between the exonuclease domain of WRN and Ku, with a particular focus on the SAP domain of Ku70, clarifying the specificities of this protein-protein interaction and allowing the exploration of its functional implications. A key conceptual conclusion was that WRN binds Ku on the end-distal side of the heterodimer.

*The Ku70 SAP domain stimulates WRN exonuclease activity* in vitro *by optimizing its positioning towards the substrate*. In nuclear extracts, Ku was identified as a partner of WRN that had no effect on the ATPase or helicase WRN activities but stimulated WRN exonuclease activity in vitro^16,18^. Ku also broadened WRN’s substrate specificity to include DNA containing oxidized purines^19,56^ that could not be removed by WRN alone^57^. Deletion experiments mapped the interaction necessary for this stimulation to Ku70 and the N-terminal region of WRN^19,56^. Our study further unveiled close contacts between the WRN N-terminal region and the Ku70 SAP domain, as well as with a Ku80 site outside the A-KBM-binding site. Indeed, our experiments with purified proteins emphasized a key functional role for the Ku70 SAP-WRN N-terminus interaction in the Ku-mediated stimulation of WRN exonuclease activity. In addition, we showed that the integrity of this interaction is necessary for the optimal recruitment of the WRN-exo fragment to laser-induced DNA damage in cells, but not of full-length WRN to replication forks. This difference could be explained by the different structures of the DNA intermediates, the contribution of additional WRN domains and/or the presence of specific interaction partners. Our site-specific mutagenesis approach points to the K596 position on the Ku70 SAP domain, K7 on Ku80, and E173, R149 on WRN as crucial for the functionality of Ku70 SAP: WRN-exo interaction. Our data with purified proteins suggest that the molecular basis of Ku-mediated WRN exonuclease stimulation may be through favoring the proximity of the WRN-exo catalytic domain and the 3’ DNA end positioned inward relative to Ku. This may explain why Ku widens WRN exonuclease substrate preference beyond a 3’ recessed strand, as substrates without Ku may be less accessible to the WRN nuclease active site and/or may result in less processive WRN nuclease activity.

*The Ku70 SAP domain controls WRN exonuclease activity at reversed replication forks*. Ku binds collapsed and reversed forks^29–37^. WRN is a major Ku interactor in cells^10–13^, and it is found among the most enriched proteins at HU-stalled forks^41^. WRN helicase activity^42,44^, along with a non-catalytic function of WRN^45^ have been implicated in the resolution of collapsed or reversed replication forks. Moreover, WRN exonuclease activity restrains fork degradation^43^. A recent study reported that rodent cells expressing a Ku80 mutant unable to bind A-KBM failed to process DNA breaks arising from replication stress and suggested that this failure was due to a defect in Ku interaction with WRN^38^. Here, we show further that the Ku70-WRN interaction unveiled by our structural data has a functional significance at stalled replication forks. Although impairing Ku70 SAP interaction with WRN did not reduce WRN recruitment at stalled or reversed forks, it resulted in fork degradation to an extent similar to a WRN exonuclease-deficient mutant. Altogether, our data show that Ku70 SAP-WRN interaction and its impact in WRN exonuclease activity is physiologically relevant for the metabolism of stalled replication forks. The Ku-WRN complex, together with other factors^55,58,59^, helps protect reversed replication forks against MRE11-mediated degradation (Fig. 6D).

Notably WRN can contact the vWA domain of Ku80 through either its N- or C-terminal A-KBM. Furthermore, our ITC and structural data established that the two WRN KBMs exhibit similar affinity and binding modes with Ku80. While their binding to Ku80 defines different positioning of WRN relative to the DNA substrate, only the one corresponding to the cryo-EM structure reported here may be permissive for the stimulation of WRN exonuclease activity. Therefore, the Ku70-WRN N-terminus interaction is likely a key regulator of the balance between both possible orientations of WRN on Ku80. The regulation of the balance between each configuration deserves further exploration, but may involve, for example, WRN phosphorylation by the Ku-associated DNA-PKcs that abolishes the Ku-mediated stimulation of WRN exonuclease^20^.

## Methods

### Cryo-EM sample preparation of Ku-hDNA_WRN-exo and data acquisition

KuFL was mixed with a hairpin DNA (hDNA^46^,) and WRN-exo in a 1:1.2:2 ratio. 3 µl of KuFL-hDNA:WRN-exo complex at ~3 mg/ml were applied on Holey Carbon copper grids, Quantifoil R1.2/1.3, 300 mesh, glow discharged for 60 seconds at 15 mA with a PELCO Easiglow (Ted Pella, Inc). The grids were then blotted for 3 seconds at either force −5 or 0 with filter paper and plunge-frozen in liquid ethane using a FEI Vitrobot Mark IV at 4 °C with 100% humidity. Data presented here were collected on a Titan Krios in the Department of Biochemistry, University of Cambridge. 4343 movies for the KuFL-hDNA:WRN-exo complex were collected in accurate centering mode using EPU software (Thermo Fisher). All data collection parameters are given in Supplementary Table 3.

### Isothermal calorimetry measurements

Regarding the interaction measurements between KuFL and the WRN peptides, KuFL heterodimer was thawed and dialyzed intensively at 4 °C against the buffer 20 mM Tris pH 8, 150 mM NaCl and 5 mM B-ME to remove the glycerol. Regarding the interaction measurements between KuFL-DNA with WRN-exo W18G or WRN-exo wild-type, the proteins were freshly purified and then dialyzed against the same buffer described previously. KuFL and WRN-exo variants were concentrated using Amicon Ultra-4 concentrators with 30 kDa and 10 kDa cut-off membranes respectively. They were centrifuged for 10 min, 11,766 × *g* at 4 °C before measurement. Routinely, KuFL or KuFL-hDNA (ratio 1:1.2) complexes (WT or variants) were injected into the sample cell (volume 2.2 ml) at a concentration of 10 μM (3 mg). Peptides or WRN-exo variants were loaded into the syringe (290 μl) at a concentration of at least 100 μM, a 10-fold higher concentration than the concentration of KuFL. KuFL or KuFL-hDNA present in the sample cell were titrated by automatic injections (30 injections of 10 µl) of the different peptides or WRN-exo variants. The enthalpy ΔH (in kcal.mol-1), the stoichiometry of the reaction and the association constant Ka (in M^-1^) are obtained by fitting the isotherm titration curve by a one-binding site model using Origin software (Malvern). All binding experiments are performed in duplicate at 25 °C.

### DNA exonuclease assays

DNA exonuclease assays (10 µl volume) were carried out in a reaction buffer containing 40 mM Tris-HCl (pH 8 at 25 °C), 4 mM magnesium chloride, 5 mM DTT, 0.1 mg/ml BSA, 1 mM ATP and 0.5 nM ^32^P-labeled oligonucleotide-based DNA substrate (in molecules). The reactions were assembled on ice and incubated at 37 °C for 1 h^16^. Next, 0.33 µl of 0.5 M EDTA and 0.66 µl of Proteinase K (20.3 mg/ml, Roche) were added to stop the reaction for 30 minutes at 50 °C. An equal amount of formamide dye (95% [vol/vol] formamide, 20 mM EDTA and bromophenol blue) was added and samples were heated at 95 °C for 4 minutes before separating them on 15% denaturing polyacrylamide gel (ratio acrylamide: bisacrylamide 19:1; Bio-Rad). After fixing in a solution containing 40% methanol, 10% acetic acid, and 5% glycerol, gels were transferred to 3 MM chromatography paper (Whatman), dried, exposed to storage phosphor screens (GE Healthcare), and scanned by Typhoon FLA 9500 (GE Healthcare).

### Multiphoton laser micro-irradiation

Live cell microscopy and multiphoton laser micro-irradiation were conducted as previously described^23^.

### In situ proximity ligation assay (PLA)

Cells were cultured onto 8-well chamber-slides (Millicell Sigma-Aldrich) and treated for 4 hours with 100 μM IAA. Subsequently, cells were labeled with 100 μM IdU and treated or not with 50 nM CPT in the presence of IAA for 1 h. After treatment, cells were preextracted in 0.5% Triton X-100 for 8 min on ice, fixed with 3% PFA, 2% sucrose in PBS 1X for 15 min at room temperature (RT), permeabilized in 0.25% Triton X-100 for 15 min at RT and blocked in blocking buffer. The in-situ proximity ligation assay (PLA) was performed using the NaveniFlex (Navinci diagnostics) Kit with anti-Mouse PLUS and anti-Rabbit MINUS PLA Probes, according to the manufacturer’s instructions. To detect proteins, we used rabbit anti-WRN (Abcam, 1:400), mouse anti-IdU antibody (Becton Dickinson, 1:100), and rabbit anti-RAD51 (Abcam AB133534, 1:200) antibodies.

### In situ protein interaction with nascent DNA replication forks (SIRF)

Exponential growing cells were seeded onto microscope chamber slide. The day of the experiment, cells were incubated with 125 μM 5-ethynyl-2’-deoxyuridine (EdU) for 8 minutes and treated with 2 mM HU for 2 hours or 50 nM CPT for 1 h. Afterwards, cells were preextracted in 0.5% TritonX-100 for 8 minutes on ice and fixed with 3% PFA, 2% sucrose in PBS 1X for 15 minutes at RT. Cells were then permeabilized in 0.25% TritonX-100 for 15 minutes at RT. To detect EdU the Click-iT™ EdU Alexa Fluor™ Imaging Kit (Invitrogen) using 5 µM Biotin-Azide was used for 30 minutes at RT. Cells were blocked in blocking buffer (Navinci diagnostics Kit). After washing with PBS, cells were incubated with the following primary antibodies: rabbit anti-WRN (Abcam, 1:400 for mAID cells or 1:2000 for nucleofected cells) and mouse anti-biotin (Abcam, 1:1000).

### DNA fiber analysis

To evaluate fork degradation cells were pulse-labeled with 50 μM CldU and then labeled with 250 μM IdU with or without 50 nM CPT treatment. When indicated, mirin was added together with IdU, as in the experimental scheme. To evaluate fork recovery cells were pulse-labeled with 50 μM CldU and then were treated or not with 50 nM CPT for 1 hour or 2 mM HU for 2 hours. Afterwards, cells were labeled with 250 μM IdU. For immunodetection of labeled tracks, the following primary antibodies were used: rat anti-CldU/BrdU (Abcam AB6326, 1:50) and mouse anti-IdU/BrdU (Becton Dickinson 347580, 1:10). Images were acquired randomly from fields with untangled fibers using an Eclipse 80i Nikon Fluorescence Microscope, equipped with a Virtual Confocal (ViCo) system. The length of labeled tracks was measured using the Image-Pro-Plus 6.0 software. A minimum of 30 individual fibers were analyzed for each experiment. In dot plots, the mean of at least three independent experiments is presented. Data from HU-treated cells for fork recovery evaluation derive from a single replicate. The value of the IdU tract length is reported in micrometers.

### Statistical analysis

Experiments shown are representative of at least three independent biological replicates unless otherwise indicated in the figure legend.

Significance for PLA, SIRF, and fibers assays was assessed using the built-in tools in Prism 10 (GraphPad Inc.) by one-way ANOVA with Tukey’s test for multiple comparisons. *P* < 0.05 was considered as significant.

### Reporting summary

Further information on research design is available in the Nature Portfolio Reporting Summary linked to this article.

## Supplementary information

Supplementary Information

Reporting Summary

Transparent Peer Review file

## Data Availability

The Cryo-EM structure data for Ku-hDNA:WRN-exo have been deposited at the PDB and EMDB with the accession codes: 9HZG and EMD-52524 [https://www.ebi.a c.uk/pdbe/entry/emdb/EMD-52524]. Source data are available at Figshare (10.6084/m9.figshare.31742572). Source Data are provided with this paper.
