# Supplementary material for: Structural basis of Ku-mediated activation of WRN exonuclease activity
Source: Nat Commun. 2026 May 13;17:6369. doi: 10.1038/s41467-026-71888-w (PMC13377182; doi:10.1038/s41467-026-71888-w)
Supplement: Supplementary file 1 — Supplementary Information [file 41467_2026_71888_MOESM1_ESM.pdf]

SUPPLEMENTARY INFORMATIONS

Structural basis of Ku-mediated activation of WRN exonuclease activity

Sayma Zahid*[1,2], Jeanne Chauvat*[3], Ilaria Ceppi*[4], Floriana Cappiello*[5], Benedetta Perdichizzi*[5], Philippe Frit[3], Dennis Gomez[3], Steven W. Hardwick[6], Pierre Legrand[2], Julien Karazi[1], Sonia Baconnais[7], Gérard Pehau-Arnaudet[8], Sébastien Britton[3], Jean-Baptiste Charbonnier[1], Amanda K. Chaplin[#6,9], Pietro Pichierri[#5], Petr Cejka[#4], Patrick Calsou[#α3], Virginie Ropars[#α1]

1. Université Paris-Saclay, CEA, CNRS, Institute for Integrative Biology of the Cell (I2BC), 91198, Gif-sur-Yvette, France.
2. Synchrotron SOLEIL, l'Orme des Merisiers, Saint Aubin, France.
3. Institut de Pharmacologie et Biologie Structurale, IPBS, Université de Toulouse, CNRS, UPS, Toulouse, France.
4. Institute for Research in Biomedicine, Università della Svizzera italiana (USI), Faculty of Biomedical Sciences, Bellinzona, Switzerland.
5. Mechanisms, Biomarkers and Models Section, Genome Stability Group, Department of Environment and Health, Istituto Superiore di Sanità, Viale Regina Elena 299, 00161 Rome, Italy.
6. Cryo-EM Facility, Department of Biochemistry, University of Cambridge, Sanger Building, Tennis Court Road, Cambridge CB2 1GA, United Kingdom.
7. Genome Integrity and Cancer UMR 9019 CNRS, Université Paris-Saclay, Gustave Roussy 114 rue Edouard Vaillant, 94805 Villejuif, France.
8. UTECH UBI, Institut Pasteur and CNRS UMR 3528, Paris, France.
9. Leicester Institute for Structural and Chemical Biology, Department of Molecular and Cell Biology, University of Leicester, Leicester, United Kingdom.

[*] These authors contribute equally to this work.
α These authors jointly supervised this work.
[#:] Corresponding authors:
Amanda K. Chaplin (ac853@leicester.ac.uk); Pietro Pichierri (pietro.pichierri@iss.it); Petr Cejka (petr.cejka@irb.usi.ch); Patrick Calsou (calsou@ipbs.fr) and Virginie Ropars (virginie.ropars@cea.fr)

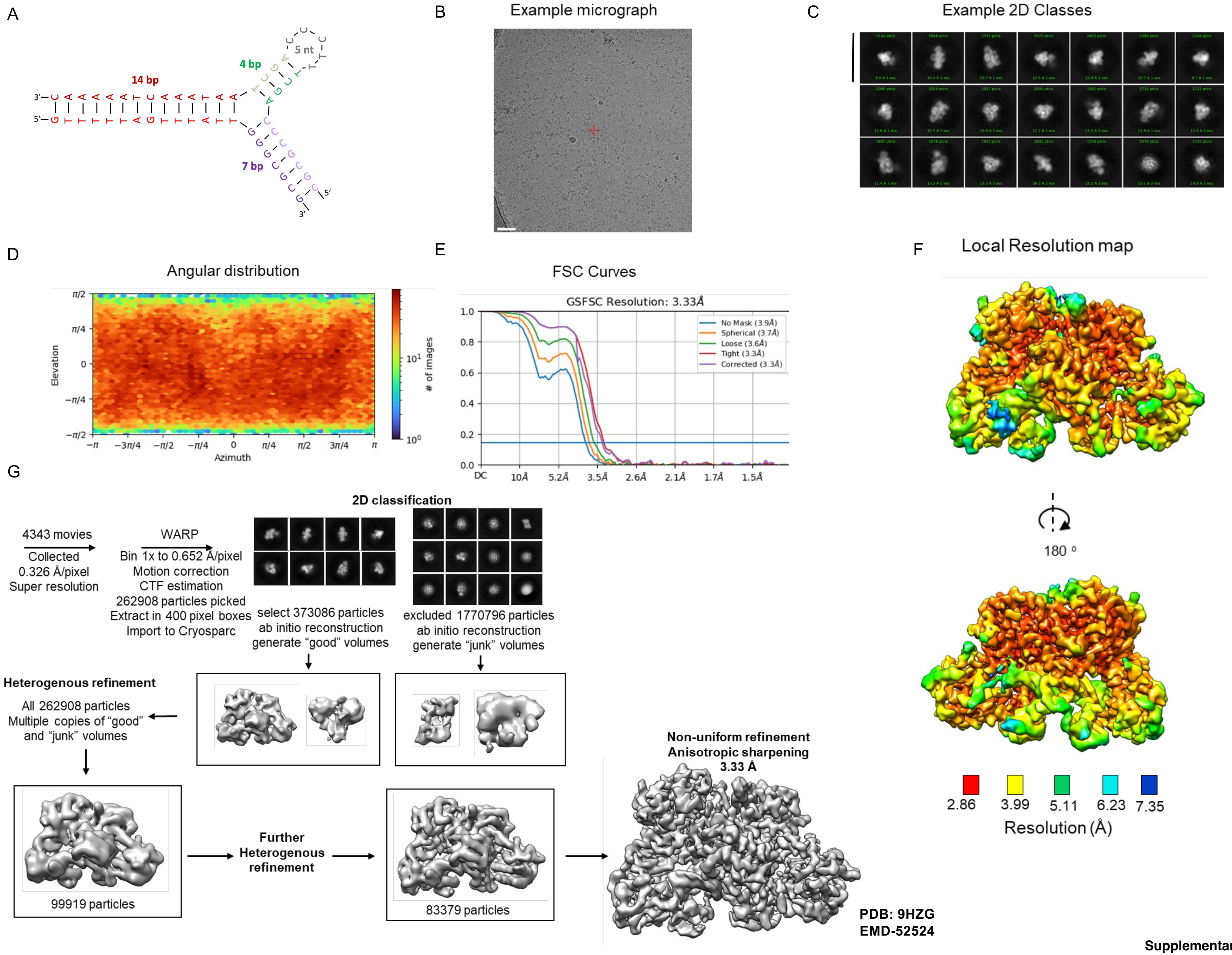

Supplementary Figure 1

Supplementary Figure 1. Single-particle cryo-EM image processing workflow for the Ku70/80-hDNA:WRN-exo complex.

A Secondary structure of the hairpin DNA (hDNA) used for cryo-EM experiments.

B A representative micrograph of Ku70/80-hDNA:WRN-exo complex recorded on a Titan Krios microscope. Scale bar 50 nm

C Representative 2D class averages of Ku70/80-hDNA:WRN-exo particles_ Scale bar 130Å

D Angular distribution calculated in cryoSPARC for particle projections. Heat map shows number of particles for each viewing angle

E Gold-standard FSC curves calculated after cryoSPARC non-uniform refinement and viewing distribution plot.

F Local resolution map of the Ku-hDNA with WRN cryo-EM map. The colours corresponding to each resolution are displayed on the specific key chart below the maps.

G Cryo-EM image processing workflow used to determine the structure of Ku70/80-hDNA:WRN-exo complex. Schematic representation of particle picking from collected movies using Warp and processing including 2D classification and ab initio reconstruction using cryoSPARC. Upon ab initio reconstructions, multiple rounds of heterogenous refinement followed. The map from the initial heterogeneous refinement is shown, followed by the final non-uniform (NU) refinement map.

A

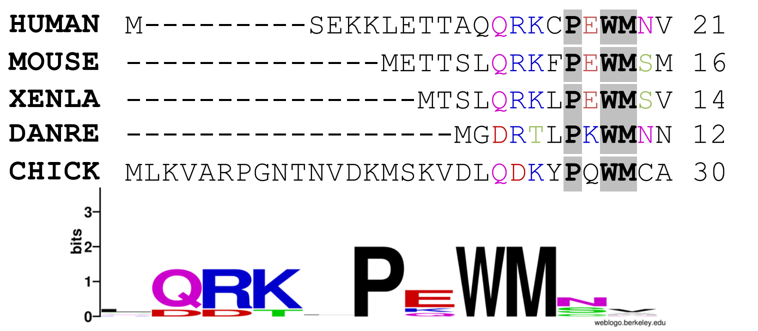

B

Peptide	Sequences	Residue range
pWRN nA (16 Aa)	Ac-TTAQQRKCPEWMNVQN-NH_2	8-23
pWRN cA (15 Aa)	Ac-SSAERKRRLPVWFAK-NH_2	1399-1413
pWRN cX (15 Aa)	Ac-SKKLMDKTKRGGLFS-OH	1418-1432
pWRN cAX (34 Aa)	Ac-SSAERKRRLPVWFAKGSDTSKKLMDKTKRGGLFS-OH	1399-1432

C

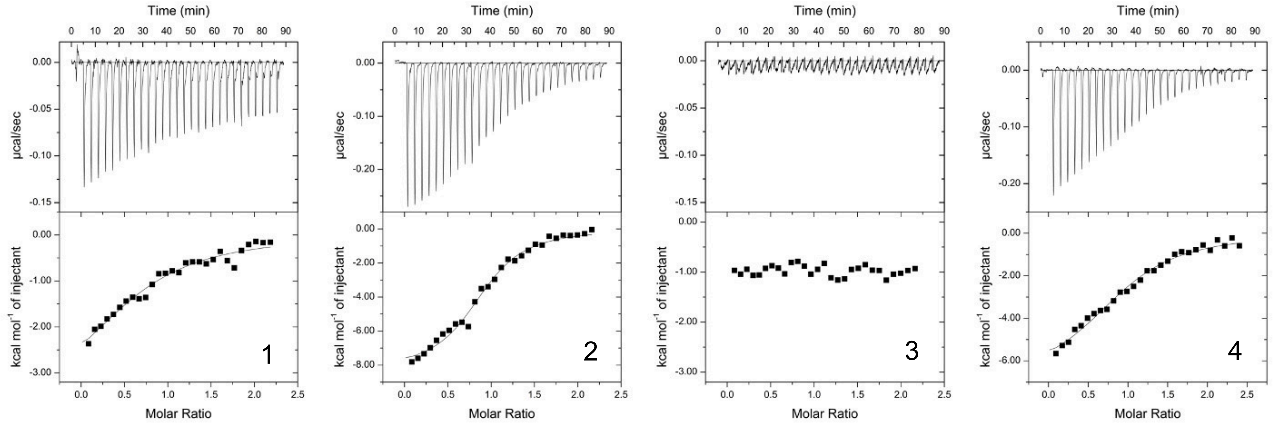

D

	ITC cell	ITC syringe	Kd (µM)
1	KuFL	WRN nA	2.8 ± 0.6
2	KuFL	WRN cA	0.5 ± 0.1
3	KuFL	WRN cX	NI
4	KuFL	WRN cAX	1.3 ± 0.3
Fig 2B	KuFL-hDNA	WRN-exo	2.0 ± 0.55
Fig 2B	KuFL-hDNA	WRN-exo W18G	NI

E F G

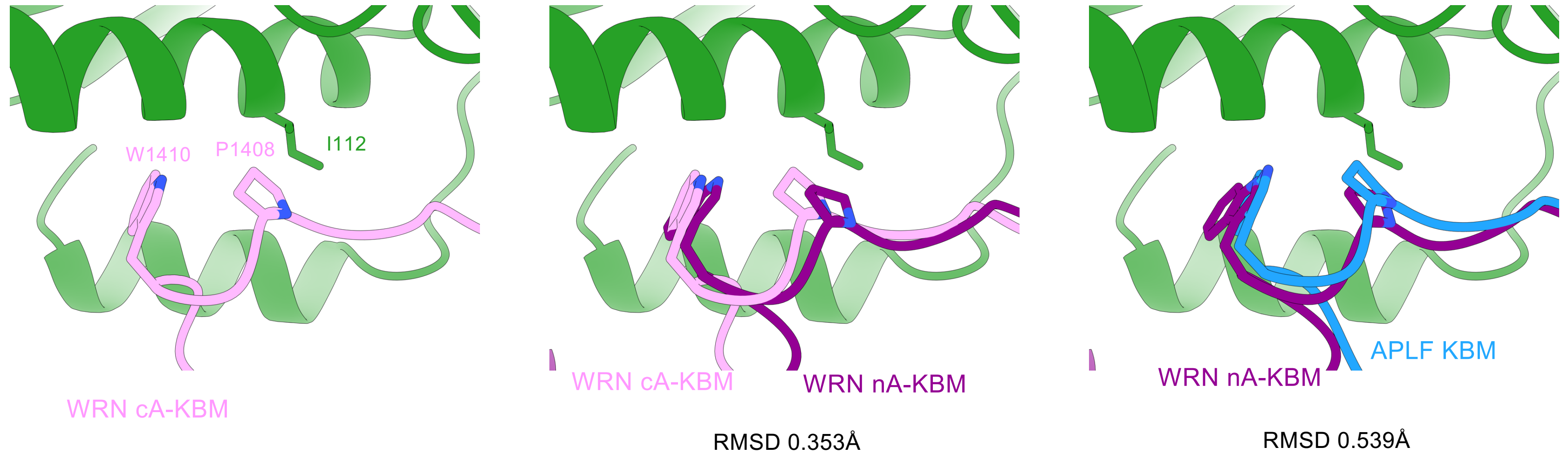

Supplementary Figure 2

Supplementary Figure 2. Interaction measurement and modeling between Ku and WRN KBM peptides.

A Top, multiple sequences alignment of WRN nA-KBM (Residue range: 1-21) showing the conserved tryptophan at position (Bold and green highlight). Bottom, logo motif of the nA-KBM (Crooks, G.E. et al., WebLogo: a sequence logo generator. Genome Res 14, 1188-90 (2004)).

B Sequences of peptides used for ITC measurements, corresponding to the three KBM regions of WRN: two APLF-like motifs (nA and cA, pink) and one XLF-like motif (cX, blue).

C ITC curves of KuFL titrated by WRN nA-KBM, cA-KBM, cX-KBM or cAX-KBM, from left to right. Source data are provided as a Source Data file.

D Dissociation constants (Kd) measured by ITC for interactions between human Ku wild-type or Ku variants (ΔSAP or K596A) and various WRN KBM peptides, WRN-exo, or the WRN-exo W18G mutant.

E Zoom of the interaction between Ku80 (green) and the WRN cA-KBM (pink) obtained from Alphafold3.

F Superimposition of the AlphaFold model of Ku80-WRN cA-KBM (light pink) with the Ku80-WRN nA (purple) cryo-EM structure, showing an RMSD of 0.35 Å between cA-KBM and nA-KBM.

G Superimposition of the Xray structure of Ku80-APLF-KBM (blue, PDB: 6ERF) with the Ku80-WRN nA (purple) cryo-EM structure, showing an RMSD of 0.54 Å between nA-KBM and APLF-KBM.

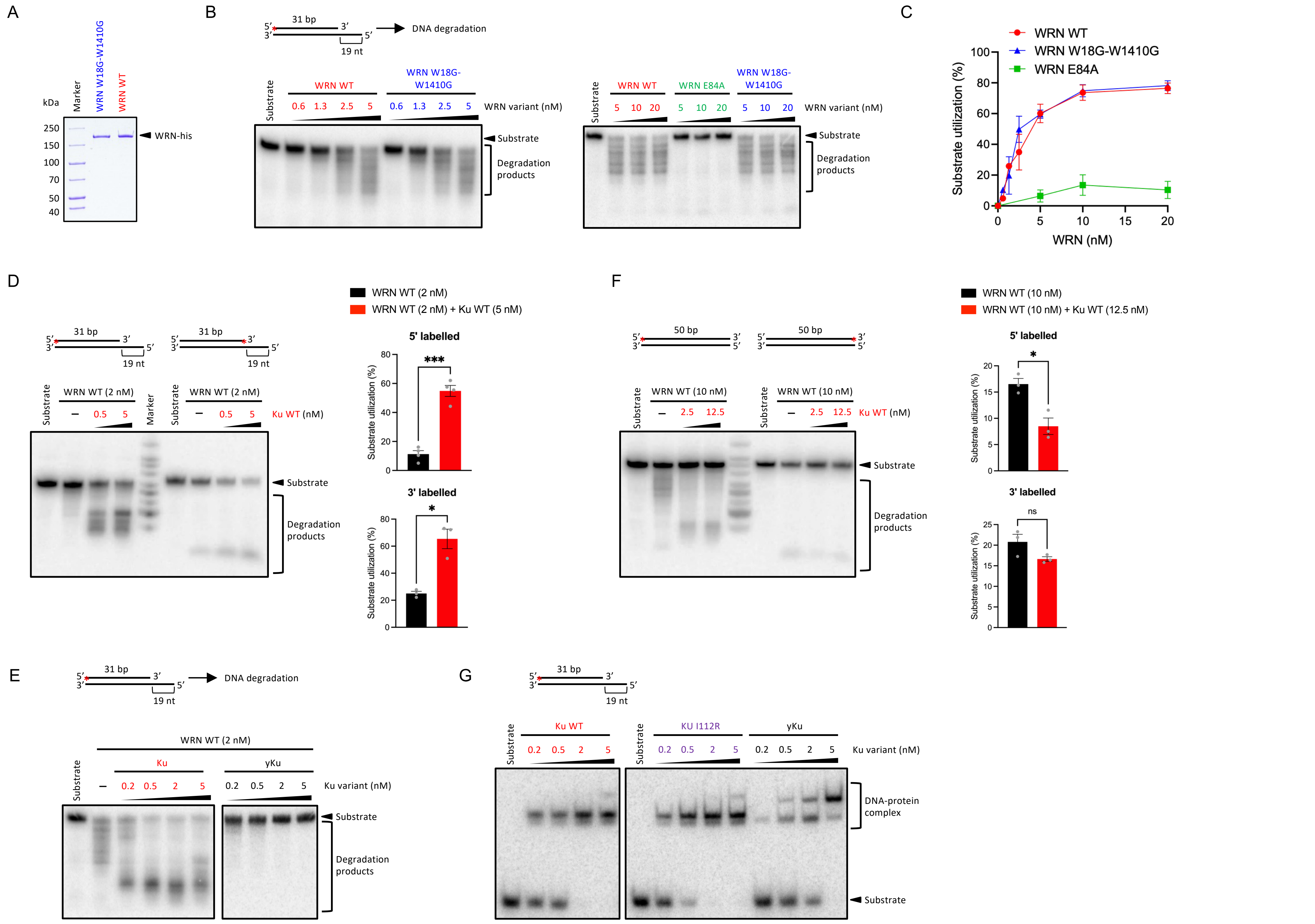

Supplementary Figure 3

Supplementary Figure 3. Measurement of Ku impact on WRN exonuclease activity with purified proteins.

A WRN variants used in this study. The polyacrylamide gel was stained with Coomassie Brilliant Blue.

B Top, schematic of the assay. Red asterisk indicates the position of the radioactive label. Bottom, representative 15% denaturing polyacrylamide gel showing the degradation of a 5′ overhang (19 nt/31 bp) DNA by WRN variants.

C Quantification of overall substrate utilization from experiments such as shown in B. Average shown; n=3 independent experiments; error bars, SEM.

D Top left, schematic of the substrates. Red asterisk indicates the position of the radioactive label. Bottom left, representative 15% denaturing polyacrylamide gel showing the degradation of differently labelled 5′ overhang (19 nt/31 bp) DNA substrates by WRN WT in the absence or presence of human Ku. Top right, quantification of overall 5'-labelled substrate utilization from experiments such as shown on the left. Bottom right, quantification of overall 3'-labelled substrate utilization from experiments such as shown on the left. Average shown; n=3 independent experiments; error bars, SEM. Statistical analysis (unpaired *t*-test): ∗p = 0.0258, ∗∗∗p = 0.0002.

E Representative 15% denaturing polyacrylamide gel showing the degradation of a 5′ overhang (19 nt/31 bp) DNA by WRN WT in the presence of human or yeast Ku. Red asterisk indicates the position of the labeling.

F Top left, schematic of the substrates. Red asterisk indicates the position of the radioactive label. Bottom left, representative 15% denaturing polyacrylamide gel showing the degradation of differently labelled dsDNA (50 bp) substrates by WRN WT in the absence or presence of human Ku. Top right, quantification of overall 5'-labelled substrate utilization from experiments such as shown on the left. Bottom right, quantification of the 3'-labelled substrate from experiments such as shown on the left. Average shown; n=3 independent experiments; error bars, SEM. Statistical analysis (unpaired *t*-test): ns = 0.1382 (not significant), ∗p = 0.0174.

G Representative 4% native polyacrylamide gel showing the electro mobility shift of a 5′ overhang (19 nt/31 bp) DNA substrate by the indicated Ku variants. n=2 independent experiments. Red asterisk indicates the position of the radioactive label.

A-G : Source data are provided as a Source Data file.

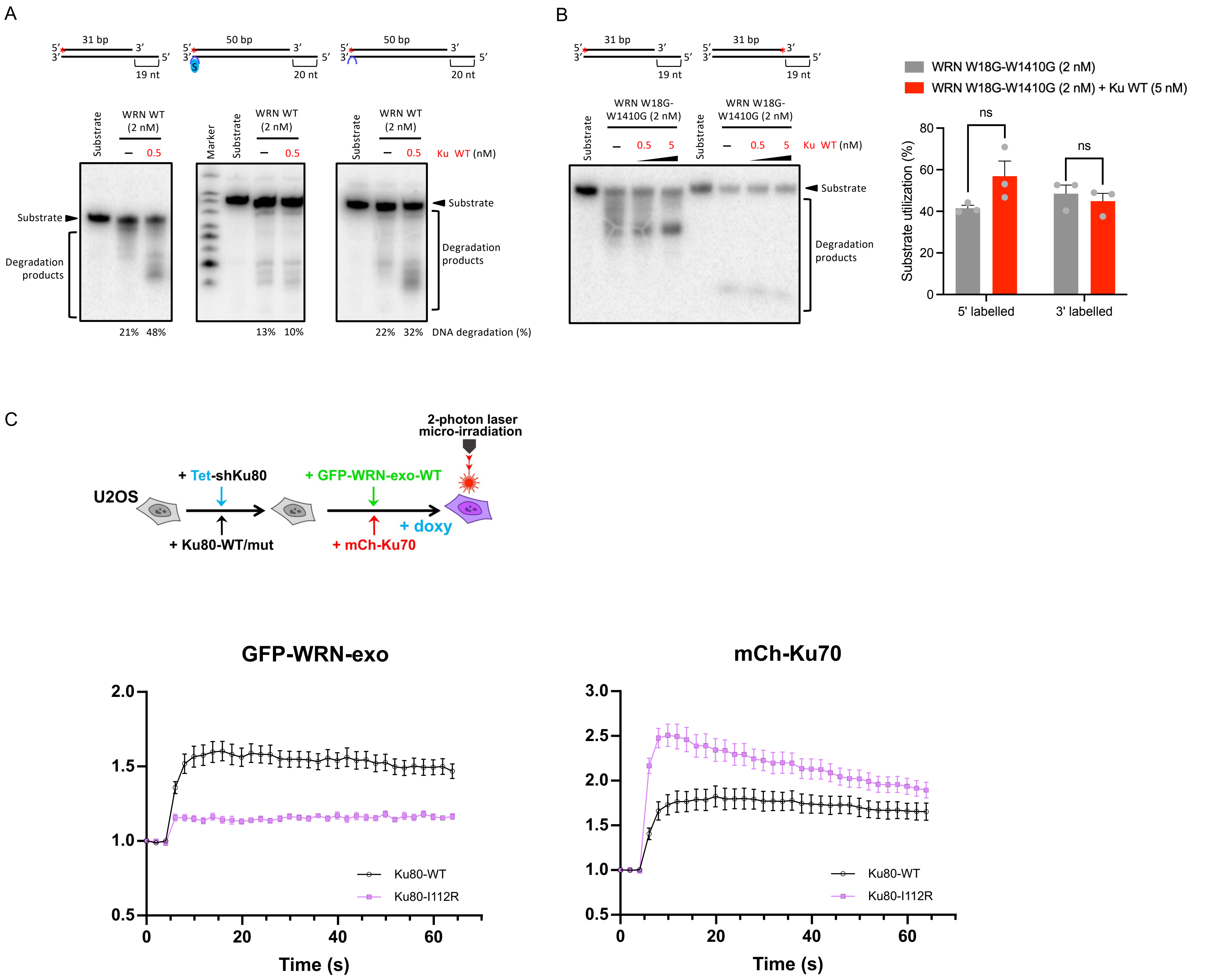

Supplementary Figure 4

Supplementary Figure 4. Measurement of Ku impact on WRN exonuclease activity with purified proteins and live recruitment of WRN-exo to DNA damage sites.

A Top, schematic of the substrates. Red asterisk indicates the position of the radioactive label. Bottom, representative 15% denaturing polyacrylamide gels showing the degradation of different 5′overhang DNA substrates either with free ends or streptavidin-blocked 3′ end (blue dot) by WRN WT in the absence or presence of human Ku.

B Top left, schematic of the substrates. Red asterisk indicates the position of the radioactive label. Bottom left, representative 15% denaturing polyacrylamide gel showing the degradation of differently labelled 5′ overhang (19 nt/31 bp) DNA substrates by WRN W18G-W1410G mutant in the presence of human Ku. Right, quantification of overall substrate utilization from experiments such as shown on the left. Average shown; n=3 independent experiments; error bars, SEM. Statistical analysis (unpaired *t*-test): ns = 0.1636 (not significant), ns = 0.5506 (not significant).

C Top, principle of the laser micro-irradiation experiment. U2OS cells engineered for Ku80 knockdown upon docycyclin addition (doxy), rescued with WT or mutant Ku80 and co-expressing GFP-WRN-exo and mCherry-Ku70, were micro-irradiated and accumulation of each fluorescence was analyzed. Bottom, results are plotted as mean values of 16 nuclei (WT Ku80) and 19 nuclei (I112R mutant) ± SEM.

A-C: Source data are provided as a Source Data file.

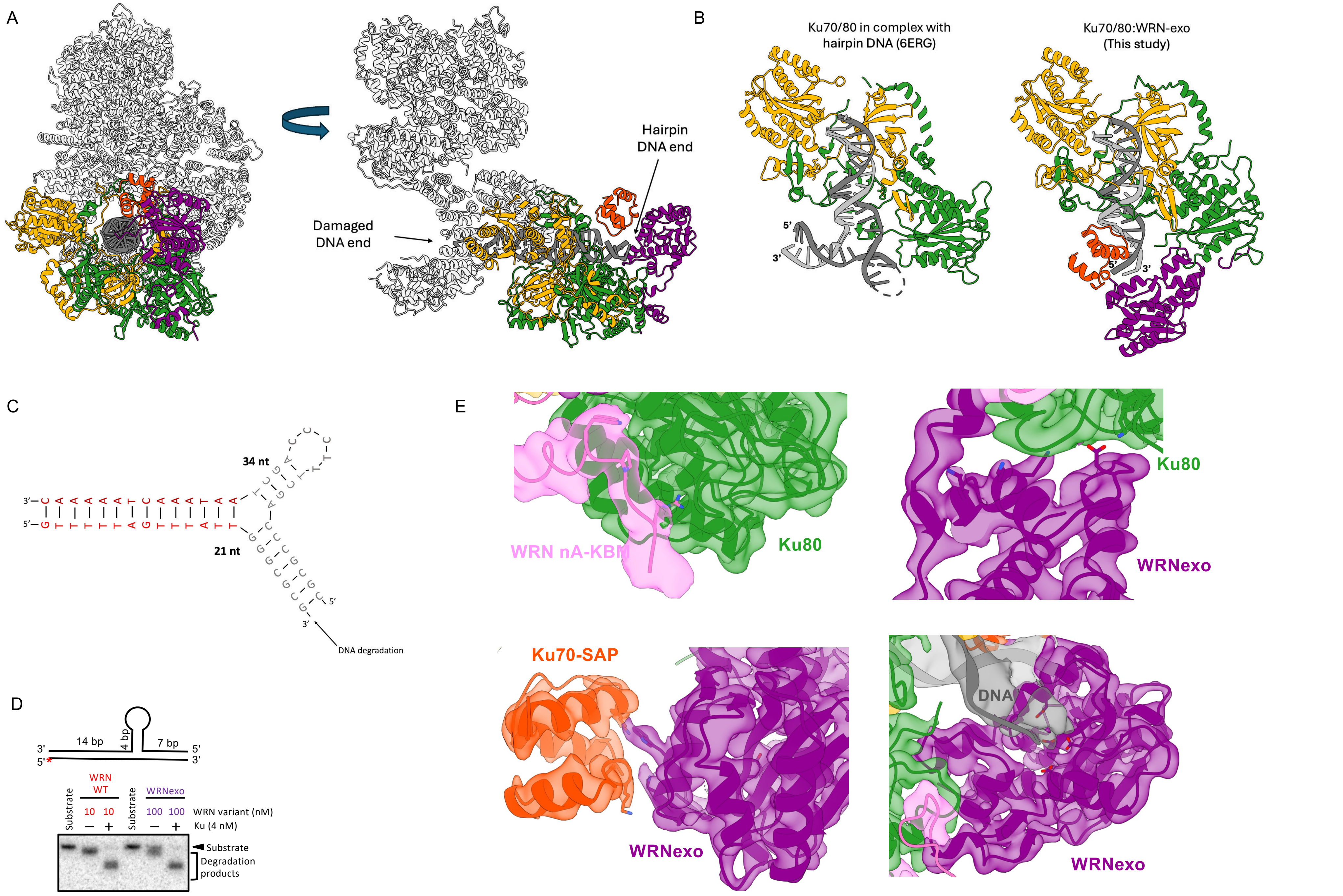

Supplementary Figure 5

Supplementary Figure 5: **Fate of the hairpin DNA in the Ku-hDNA:WRN-exo complex.**

A Superposition of Ku70/80-hDNA:WRN-exo structure onto the monomeric DNA-PK holoenzyme from PDB 6ZHA showing the WRN exonuclease domain (purple) positioned away from the DNA damage site. DNA-PKcs is shown in grey, Ku70/Ku80 in orange and green, respectively.

B Comparison of the solved hairpin DNA structure in Ku70/80-hDNA:WRN-exo with the structure from PDB 6ERG.

C Secondary structure of the hairpin DNA used for the cryo-EM data. The nucleotides colored in gray are not visible in the cryoEM map.

D Top, schematic of the substrate. Red asterisk indicates the position of the radioactive label. Bottom, representative 15% denaturing polyacrylamide gel showing the degradation of the hairpin DNA substrate used in cryoEM by WRN WT or WRN-exo construct in the presence of Ku. Source data are provided as a Source Data file.

E Segments of the cryo-EM map in different regions fitted with the Ku70/80-hDNA:WRN-exo model. Top left: nA-KBM of WRN (pink) bound to the vWA domain of Ku80. Top right, exonuclease domain of WRN (purple) interacting with Ku80. Bottom left, SAP domain of Ku70 (orange) interacting with the exonuclease domain of WRN (purple). Bottom right, overall view of the WRN exonuclease domain and its active site with hDNA (grey).

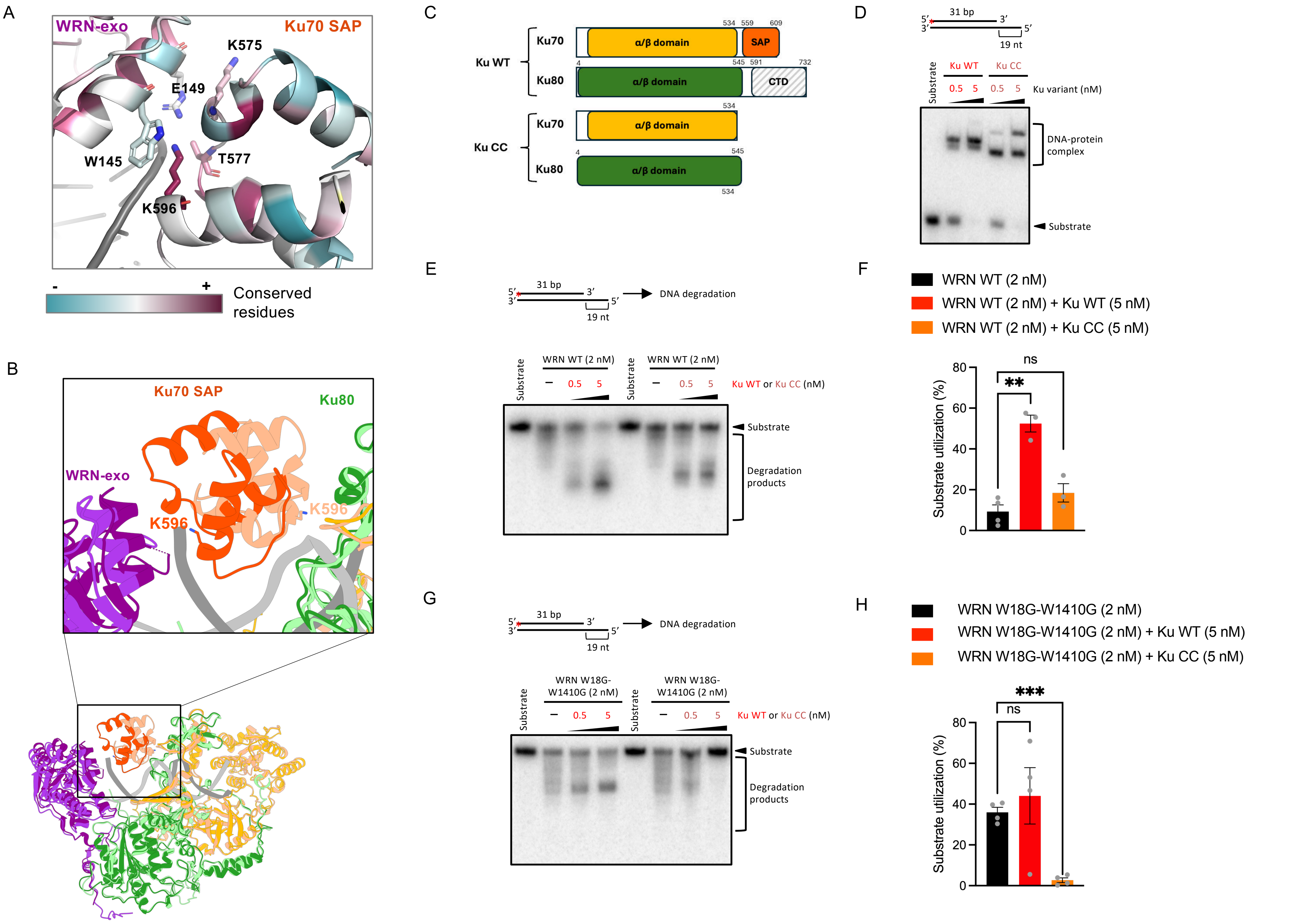

Supplementary Figure 6

Supplementary Figure 6: **Ku C-ter domains contribute to stimulation of WRN exonuclease by Ku.**

A Consurf models of Ku70/80 and WRN-exo showing the highly conserved residues in brown and variable residues in blue.

B Alphafold3 model of Ku-hDNA:WRN-exo (light color) superimposed to the cryoEM structure. A zoom-in of the Ku70 SAP domain illustrates the disrupted interaction between WRN-exo (purple) and the residue K596 of Ku70 (orange) in the AlphaFold model.

C Schematic representation of Ku70/80 (light orange/green) constructs used in the following experiments.

D Top, schematic of the substrate. Red asterisk indicates the position of the radioactive label. Bottom, representative 4% native polyacrylamide gel showing the electromobility shift of a 5′ overhang (19 nt/31 bp) DNA substrate by the indicated Ku variants. n=3 independent experiments.

E Top, schematic of the assay. Red asterisk indicates the position of the radioactive label. Bottom, representative 15% denaturing polyacrylamide gel showing the degradation of a 5′ overhang (19 nt/31 bp) DNA by WRN WT in the presence of human Ku WT or CC mutant.

F Quantification of overall substrate utilization from experiments such as shown in E. Average shown; n=3 independent experiments; error bars, SEM. Statistical analysis (unpaired *t*-test): ns = 0.1757 (not significant), $**p = 0.0010$.

G Top, schematic of the assay. Red asterisk indicates the position of the radioactive label. Bottom, representative 15% denaturing polyacrylamide gel showing the degradation of a 5′ overhang (19 nt/31 bp) DNA by WRN W18G-W1410G mutant in the presence of human Ku WT or CC mutant.

H Quantification of overall substrate utilization from experiments such as shown in G. Average shown; n=4 independent experiments; error bars, SEM. Statistical analysis (unpaired *t*-test): ns = 0.6032 (not significant), $***p = 0.0002$.

E-H: Source data are provided as a Source Data file.

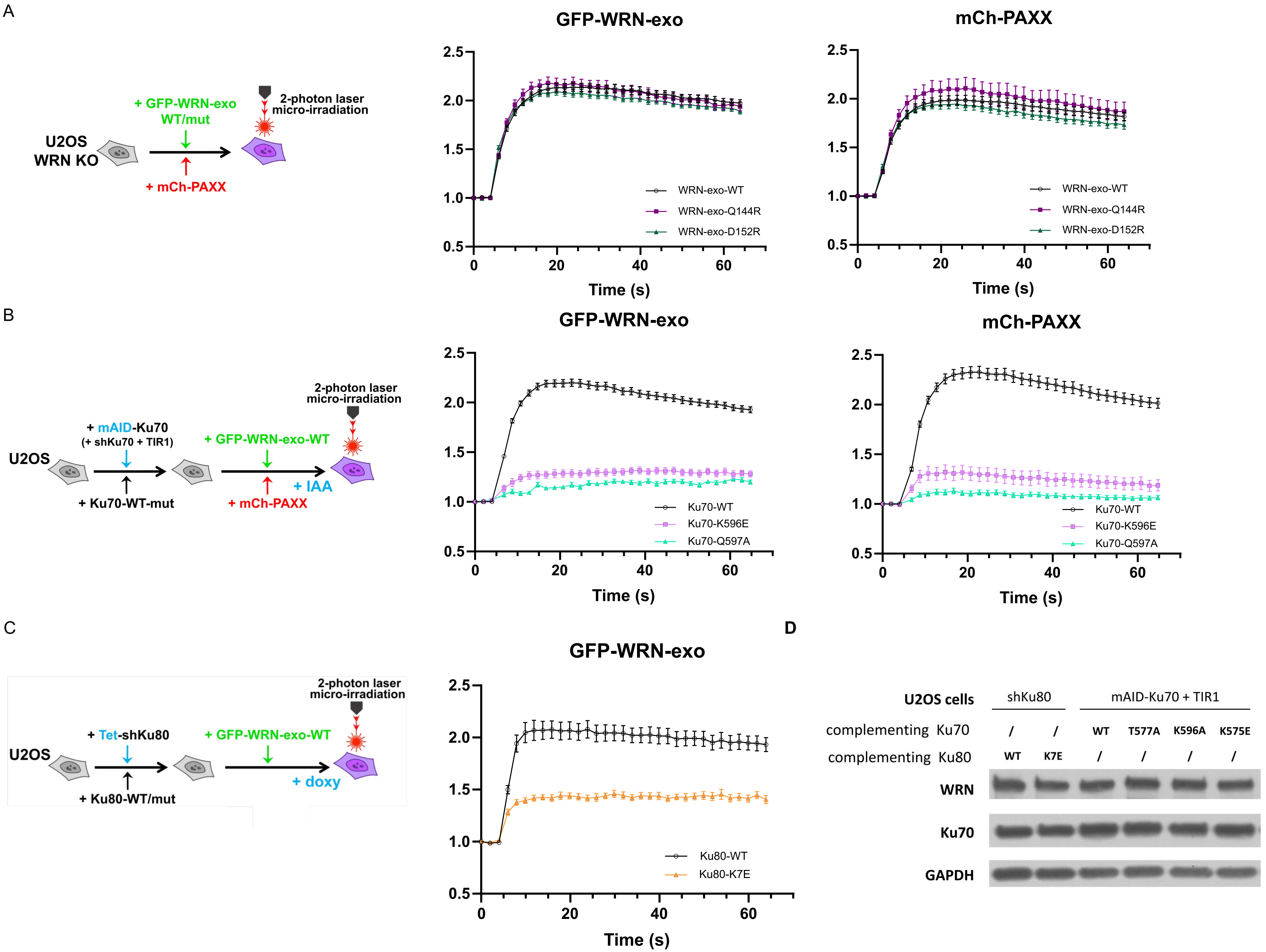

Supplementary Figure 7

Supplementary Figure 7: **Live recruitment of WRN-exo to DNA damage sites.**

A Left, principle of the laser micro-irradiation experiment. U2OS cells knocked-out for WRN and coexpressing WT or mutant GFP-tagged WRN-exo and mCherry-tagged PAXX, were micro-irradiated and accumulation of each fluorescence was analyzed. Right, quantification of fluorescence accumulation at laser-induced DNA damage sites of GFP-WRN-exo and mCherry-PAXX in U2OS WRN KO cells. Right: results are plotted as mean values of at least 57 nuclei (WT WRN-exo), 52 nuclei (Q144R mutant) and 55 nuclei (D152R mutant) ± SEM.

B Left, principle of the laser micro-irradiation experiment. U2OS cells engineered for auxin (IAA)-induced Ku70 knockdown, rescued with WT or mutant Ku70 and coexpressing GFP-WRN-exo and mCherry-tagged PAXX, were micro-irradiated and accumulation of each fluorescence was analyzed (Right part). Right, results are plotted as mean values of 130 nuclei (WT Ku70), 44 nuclei (K596E mutant) and 45 nuclei (Q597A mutant) ± SEM.

C Left, principle of the laser micro-irradiation experiment. U2OS cells engineered for Ku80 knockdown upon doxycylin addition (doxy), rescued with WT or mutant Ku80 and expressing GFP-WRN-exo were micro-irradiated and accumulation of fluorescence was analyzed. Right, results are plotted as mean values of 20 nuclei (WT Ku80) and 18 nuclei (K7E mutant) ± SEM.

D Western blotting analysis showing the expression of Ku and WRN proteins. U2OS cells engineered for auxin (IAA)-induced Ku70 knockdown were rescued with WT or mutant Ku70 or Ku80. Equal protein loading was checked using GAPDH antibody as loading control.

A-D: Source data are provided as a Source Data file.

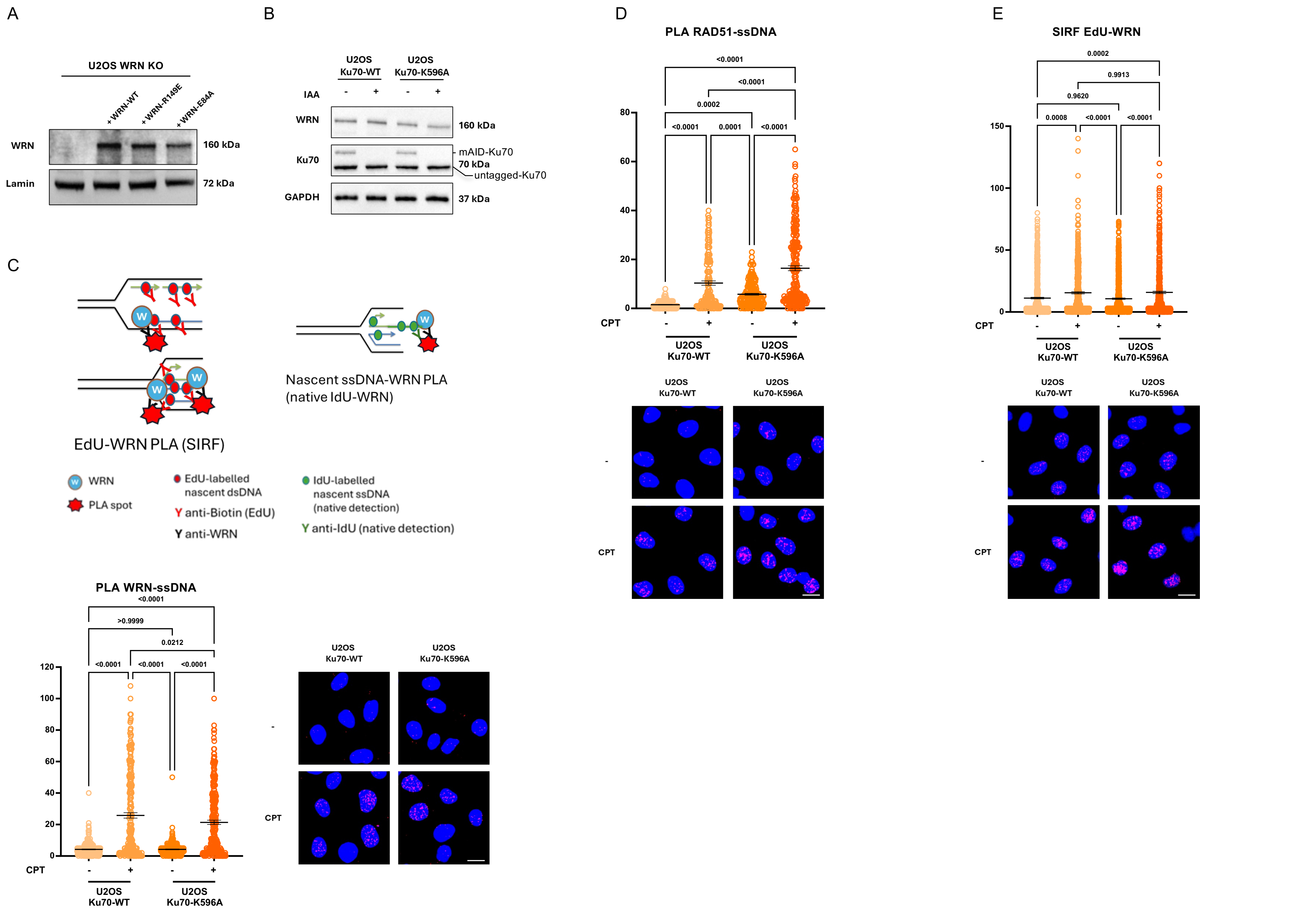

Supplementary Figure 8

Supplementary Figure 8. Impact of Ku-WRN interaction on resection at stalled replication forks.

A Western blotting analysis showing the expression of WRN protein. Nucleofected U2OS WRN $^{-/-}$ cells were evaluated for WRN wild type, WRN R149E or WRN E84A expression. Equal protein loading was checked using Lamin antibody as loading control.

B Western blotting analysis showing the expression of Ku70 protein. U2OS Ku70-WT and U2OS Ku70-K596A cells were treated with IAA for 4 h to replace Ku70-mAID (upper band) by untagged Ku70 WT or K956A (lower band). Equal protein loading was checked using GAPDH antibody as loading control.

C Top, schematic representation of the difference between Edu-WRN PLA (SIRF WRN) and nascent ssDNA-WRN PLA (native IdU-WRN) assays. In situ PLA between WRN and nascent ssDNA in U2OS cells expressing Ku70-WT or Ku70-K596A. After 4 h IAA treatment, cells were labelled with IdU and treated with or without CPT. Bottom left, PLA spots derive from at least 250 cells (three independent experiments). Bars represent mean ± SEM. Bottom right, representative images (scale bar: 20 μm).

D Top, in situ PLA between RAD51 and nascent ssDNA in U2OS cells expressing Ku70-WT or Ku70-K596A. After 4 h IAA treatment, cells were labelled with IdU and treated with or without CPT. PLA spots derive from at least 160 cells (three independent experiments). Bars represent mean ± SEM. Bottom, representative images (scale bar: 20 μm).

E Top, SIRF PLA analysis of WRN localization at EdU-labelled DNA in U2OS cells expressing Ku70-WT or Ku70-K596A. PLA spots derive from at least 530 cells (three independent experiments). Bars represent mean ± SEM. Bottom, representative images (scale bar: 20 μm).

A-E: Source data are provided as a Source Data file.

Supplementary Table 1. Oligonucleotides used for site-directed mutagenesis of WRN and Ku constructs, and CRISPR guide for WRN

Oligonucleotide Name	Primer Sequence (5′ to 3′)
WRN_W18G_F	GCACAGCAGCGGAAATGTCCTGAAGGGATGAATGTGCAGAATAAAAGATG
WRN_W18G_R	CATCTTTTATTCTGCACATTCATCCCTTCAGGACATTTCCGCTGCTGTGC
WRN_W1410G_F	GAGAAAGAGACGATTACCTGTGGGGTTTGCCAAAGGAAGTGATACCAG
WRN_W1410G_R	CTGGTATCACTTCCTTTGGCAAACCCCACAGGTAATCGTCTCTTTCTC
Guide CRISPR WRN n°2	AAACCAGTACAGGTGATCTG
Guide CRISPR WRN n°4	ATCCTGTGGAACATACCATG
Kpn2-Ku70-F	CCTCGAGGTTTAAACTACGGgatcTCCGGAgccacc
Mlu-Ku70-F	CGGCATGGACGAGCTGTACAAGACGCGTATGTCAGGGTGGGAGTCATATTAC AAAACC
Bcu-Ku70-R	CCAGAGGTTGATTATCATATGACTAGTTCAGTCCTGGAAGTGCTTGGTGAGGG CTTC
Ku70-K575E-F	CAGCAAGGGTACGCTGGGCgAGTTCACTGTGCCCATGCTGAAAG
Ku70-K575E-R	CTTTCAGCATGGGCACAGTGAACTcGCCCAGCGTACCCTTGCTG
Ku70-T577A-F	GGGTACGCTGGGCAAGTTCgCTGTGCCCATGCTGAAAGAGGCCTGCC
Ku70-T577A-R	CATGGGCACAGcGAACTTGCCCAGCGTACCCTTGCTGATG
Ku70-K596A-F	CTTACGGGCTGAAGAGTGGgCTGAAGgcGCAGGAGCTGCTGGAAGCCCTC
Ku70-K596A-R	GAGGGCTTCCAGCAGCTCCTGCgcCTTCAGcCCACTCTTCAGCCCGTAAG
Ku70-K596E-F	CTTACGGGCTGAAGAGTGGgCTGAAGgAGCAGGAGCTGCTGGAAGCCCTC
Ku70-K596E-R	GAGGGCTTCCAGCAGCTCCTGCTcCTTCAGcCCACTCTTCAGCCCGTAAG
Ku70-Q597A-F	CGGGCTGAAGAGTGGgCTGAAGAAGgcGGAGCTGCTGGAAGCCCTCACC
Ku70-Q597A-R	GGTGAGGGCTTCCAGCAGCTCCgcCTTCTTCAGcCCACTCTTCAGCCCG
Kpn2-WRN-F	ccactgTCCGGAACCGCCGCCATGAGTGAAAAAAAATTGG
Mlu-WRN-236-R	cctcttACGCGTCCTTTGCACAGTATCATCCAAAATCTC
Kpn2-Ku80-K7E-F	ctcgtcTCCGGAATGGTGCGGTCGGGGAATgAGGCAGCTGTTGTGCTGTG
pLV-R	CCAGTCAATCTTTCACAAATTTTGTAATCCAGAGG
WRN-Q144R-F	GTAGGAATTGAAGGAGATCgGTGGAAACTTCTACGAGACTTTGATATC
WRN-Q144R-R	CAAAGTCTCGTAGAAGTTTCCACcGATCTCCTTCAATTCCTACACC
WRN-R149E-F	CAGTGGAAACTTCTAgaAGACTTTGATATCAAATTGAAGAATTTTGTGGAG

WRN-R149E-R	CTTCAATTTGATATCAAAGTCTtcTAGAAGTTTCCACTGATCTCCTTCAATTCC
WRN-R173E-F	GCTGAAATGTACAcgGACCTGGAGCCTTAACAGTCTGGTTAAAC
WRN-R173E-R	CTGTTAAGGCTCCAGGTCcgTGTACATTTCAGCTTTTTATTGGC
WRN-D152R-F	CTTCTACGAGACTTTcgTATCAAATTGAAGAATTTTGTGGAGTTGAC
WRN-D152R-R	CTTCAATTTGATAcgAAAGTCTCGTAGAAGTTTCCACTGATC
hWRN-Mlu-F	CTCTCACGCGTACCGCCGCCATGAGTGAAAAAAAATTGGAAACAACTGC
hWRN-Xma-R	CTCTGCCCGGGTTAACTAAAAAGACCTCCCCTTTTCGTTTTGTCC

Supplementary Table 2. Oligonucleotides used for DNA substrate preparation. [TB] indicates the position of the biotin.

**Name**	**Sequence (5′ to 3′)**
X12-4SC	GCGATAGTCTCTAGACAGCATGTCCTAGCAA
X12-3	GACGTCATAGACGATTACATTGCTAGGACATGCTGTCTAGAGACTATCGC
X12-4C	GCGATAGTCTCTAGACAGCATGTCCTAGCAATGTAATCGTCTATGACGTC
PC216_50nt	GTAAGTGCCGCGGTGCGGGTGCCAGGGCGTGCCCTTGGGCTCCCCGGGCG
PC209	GATGCATGAGGTGGAGTACGCGCCCGGGGAGCCCAAGGGCACGCCCTGGCACCCG CACCGCGGCACT**[TB]**AC

Supplementary Table 3. Cryo-EM data collection, refinement and validation statistics

	**Ku70/80-hDNA-WRN-exo**<br>PDB:9HZG<br>EMD-52524
**Data collection and processing**	
Detector	Gatan K3
Magnification	130k
Energy filter slit width (eV)	20
Voltage (kV)	300
Flux on detector (e/pix/sec)	12.475
Electron exposure on sample (e–/Å^2)	47.24
Target defocus range (μm)	0.7-2.5
Calibrated pixel size (Å)	0.652
Symmetry imposed	C1
Extraction box size (pixels)	400
Initial particle images (no.)	262908
Final particle images (no.)	83379
**Refinement**	
Map resolution at FSC=0.143 (Å)*	3.33
Model composition	
Non-hydrogen atoms	10561
Protein residues	1302
Nucleotides	34
B factor (Å^2)	
Protein	123.63
DNA	224.58
R.m.s deviations	
Bond lengths (Å)	0.003
Bond angles (°)	0.488
Validation	
Molprobity score	2.37
Clashscore	31.91
Poor rotamers (%)	0.3
Ramachandran plot	
Favored (%)	94.34
Allowed (%)	5.35
Disallowed (%)	0.31

SUPPLEMENTARY METHODS

Expression and Purification of WRN-exo.

The codon-optimized cDNA for expressing the N-terminal region of WRN (1-236) in *E. coli*, referred to as WRN-exo, was cloned into a pET11a vector supplied by Genscript, between the NdeI and BamHI restriction sites. WRN-exo mutants (W18G, R149E and W145A) were prepared by Genscript through mutagenesis of the corresponding pET11a-WRN-exo plasmid. For a final 800 ml culture, 20 ml of pre-culture was diluted in LB culture medium supplemented with 0.2 mg/ml of ampicillin and incubated at 37°C (OD_{600nm} of ~0.2). Once OD_{600nm} of the cell cultures reached a value between 0.6-0.8, 250 µM of IPTG (final concentration) was supplemented to the medium. The cultures were incubated at 20°C overnight. The cell cultures were arrested by centrifugation 20 minutes at 3,993 g and at 4°C. The cell pellet of 800 ml of culture was resuspended with 20 ml buffer containing 20 mM Tris-HCl pH 9.0, 150 mM KCl, 850 mM NaCl, 20 mM imidazole pH 9.0, 10 mM β-mercaptoethanol (B-ME) and flash-frozen in liquid-nitrogen for storage at -80°C. The bacterial pellets were resuspended on ice in 35 ml wash/lysis buffer containing 20 mM Tris-HCl, 150 mM KCl, 850 mM NaCl, 20 mM imidazole, 10 mM BME, 5% glycerol, 300 µg lysozyme and cocktail of protease inhibitor EDTA-Free (Roche). Cells were sonicated by ultrasound on ice for 5 cycles of 1 minute with 1 s ON/1 s OFF pulses and an increasing amplitude from 30% to 60%. Then, cells were incubated during 20 minutes on ice with 2 µl of benzonase (25 kU stock, Sigma Aldrich) and 10 mM $MgCl_2$. The lysate was then clarified by centrifugation during 30 minutes at 48,000 g at 4°C. The soluble fraction was filtered through a 0.45 µm filter to eliminate aggregates before incubation with 20 ml nickel-coated agarose beads for 1 hour at 4°C on a rotating wheel. The beads were washed with 7 column volumes (CV) of wash/lysis buffer and eluted with 5 times 10 ml (total 2.5 CV) of 20 mM Tris-HCl, 150 mM NaCl, 300 mM imidazole and 10 mM B-ME. Elution fractions of interest were pooled and dialysed against buffer Q_AWRN (20 mM Tris-HCl, 50 mM NaCl, 10 mM B-ME and 0.5 mM EDTA) overnight at 4°C using a Spectra-Por (Spectrum^{TM}) dialysis membrane with a cut-off of 6-8 kDa. The dialysed WRN protein was loaded onto the pre-equilibrated Resource Q column (6 ml, Cytiva). The column was washed with 3 CV of buffer Q_AWRN. The elution of WRN-exo was carried out by performing a gradient from 0 to 40% of buffer containing 20 mM Tris-HCl, 1 M NaCl, 10 mM BME and 0.5 mM EDTA over 12 CV. The elution fractions of interest were pooled and dialysed extensively against a freezing buffer (FB) containing 20 mM Tris-HCl, 150 mM NaCl, 5 mM B-ME, 0.5 mM EDTA and 5% glycerol, using a dialysis membrane with a cut-off of 6-8 kDa at 4°C.

Expression and Purification of recombinant proteins.

For cryo-EM, exonuclease assays and ITC, the human full-length Ku70(1-609)/Ku80(1-732) or KuCC (Ku70 (1-544)/Ku80(1-551)) or KuFL-K596A were expressed in *Sf21* insect cells using a MultiBac expression system (1). KuFL-K596A was prepared by Genscript through mutagenesis of the pFL_Ku70_Ku80 plasmid. The Ku80 subunit contains a 10x His-tag followed by a TEV protease site on its N-terminus. The Ku70/Ku80 heterodimer wild-type and variants were purified according to the protocol described in Nemoz et al., 2018 (2). WRN mutants (W18G-W1410G and WRN W18G) were prepared by mutating the respective pFB-MBP-WRN-his plasmid by QuickChange site-directed mutagenesis kit following manufacturer’s instructions (Agilent Technology). Primers used for site-directed mutagenesis are listed in Supplementary Table 1. The point mutants were expressed and purified using the same procedure as the wild-type protein (3). Briefly, WRN wild-type and WRN mutants were expressed in *Spodoptera frugiperda* 9 (*Sf*9) insect cells using bac to bac baculovirus expression system (Invitrogen) and purified by first binding to amylose resin followed by the cleavage of maltose-binding protein (MBP) tag with prescission protease and subsequently binding to nickel-NTA agarose resin (Qiagen) followed by dialysis for 2 hours (4). Yeast Ku was expressed in *Sf*9 cells and purified by affinity chromatography taking advantage of the Ku70 N-terminal MBP tag and the Ku80 C-terminal FLAG-tag (5).

DNA substrate preparation.

Oligonucleotide-based DNA substrates were radiolabeled either at the 5' terminus with [γ-^{32}P]ATP (Perkin Elmer) and T4 polynucleotide kinase (New England Biolabs), or at the 3' terminus with [α-^{32}P]dCTP (Perkin Elmer) and terminal transferase (New England Biolabs) according to the manufacturer’s instructions (3). Unincorporated nucleotides were removed using Micro Bio-Spin P-30 Tris chromatography columns (Biorad). The 5′ overhang DNA substrate was prepared by annealing of the oligonucleotides X12-4SC and X12-3, while the dsDNA substrate was prepared by annealing of the oligonucleotides X12-3 and X12-4C. The biotinylated 5′ overhang DNA substrate was prepared by annealing oligonucleotides PC216_50nt and PC209. Oligonucleotides used for DNA substrate preparation are listed in Supplementary Table 2.

The hairpin DNA substrate used for cryoEM and ITC was previously described by Walker et al., 2001 (6). The oligonucleotides of 34 and 21 nucleotides were purchased from Eurofins or Eurogentec. Lyophilized oligonucleotides were resuspended in ultrapure water and

complementary strands were mixed at a 1:1 molar ratio and a final concentration of 500 μM. Then, the mixture was heated at 90°C for 5 minutes in a microcentrifuge tube and cooled slowly overnight to room temperature.

CryoEM data acquisition, Images processing, refinement and model building.

Data presented here were collected on a Titan Krios in the Department of Biochemistry, University of Cambridge. All data collection parameters are given in Supplementary Table 3. 4343 movies for the KuFL-hDNA:WRN-exo complex were collected in accurate centering mode using EPU software (Thermo Ficher). CTF correction, motion correction, and particle picking were performed using Warp (7). 262,908 particles for KuFL-hDNA:WRN-exo picked by boxnet2 masked neural network model in Warp were imported into CryoSPARC (8) and were subjected to 2D classification, and *ab initio* reconstruction to generate an initial 3D model. Initial 3D models were subjected to multiple rounds of heterogeneous refinement to remove particles which did not represent Ku. The models' resolution and density quality were further improved by performing homogeneous refinement and finally non-uniform refinement. The classification process is summarized schematically in Figure S1G. The reconstruction at 3.33 Å of KuFL-hDNA:WRN-exo contained 83,379 particles. Final map resolutions were calculated in CryoSPARC (7) by Fourier shell correlation at 0.143 cut-off. For structure refinement, previously published crystallographic models of Ku70/80 with or without hDNA (PDB: 1JEQ, 1JEY, 6ERF) were rigid body-fitted into the final cryo-EM maps using UCSF Chimera (9). The crystallographic models of WRN exonuclease domain (PDB: 2FBT) and the N-terminal KBM of WRN (PDB: 6TYV) were manually adjusted, refined and extra residues were built using Coot (10). Several rounds of real-space refinement were then performed using PHENIX (11) until outliers were fixed.

Synthetic peptides.

The synthetic peptides containing the KBM motif were purchased from Genecust at 95% purity, and the concentrations of the peptide stock solutions were determined by amino acid composition analyses. WRN peptides used in ITC measurements are listed below:

pWRNnA Ac-TTAQQRKCPEWMNVQN-NH2

pWRNcA Ac-SSAERKRRLPVWFAK-NH2

pWRNcX Ac-SKKLMDKTKRGGLFS-OH

pWRNcAX Ac-SSAERKRRLPVWFAKGSDTSKKLMDKTKRGGLFS-OH

at 25°C.

Electrophoretic mobility shift assay.

EMSAs were performed in 15 µl volume with the same reaction buffer utilized for the exonuclease assays. After incubation at 37°C for 1 hour, 5 µl of EMSA loading dye (bromophenol blue and 50% glycerol) were added and samples were immediately loaded either on 4% native polyacrylamide gels. Gels were run on ice, transfer on 17 CHR chromatography paper (Whatman), dried, exposed to storage phosphor screens (GE Healthcare) and scanned by Typhoon FLA 9500 (GE Healthcare).

Cell lines, cell culture.

U2OS cells (human osteosarcoma cell line from a female patient (12), obtained from ECACC, Salisbury, UK) and HEK-293T human embryonic cells, were grown in Dulbecco's modified Eagle's medium (DMEM, Eurobio, France) supplemented with 10% fetal calf serum (Eurobio, France), 125 U/ml penicillin, and 125 µg/ml streptomycin. Cells were maintained at 37°C in a 5% CO_2 humidified incubator.

Cell engineering.

All oligonucleotides used (Supplementary Table 1) were purchased from Eurofins Genomics (Ebersberg, Germany). Restriction and modifying enzymes (Phusion and T4 DNA Ligase) were from ThermoFisher Scientific (Illkirch, France). All constructs were checked by sequencing (Eurofins Genomics).

The generation of U2OS cells expressing an inducible shRNA against Ku80 and the generation of U2OS cells expressing a mini-auxin-inducible degron-tagged Ku70 protein (mAID-Ku70) in place of the endogenous Ku70 protein have been previously described (13,14).

U2OS WRN KO cells were constructed by transfection via the CRISPR/Cas9 system using two guide RNAs, WRN-1: 5' AAACCAGTACAGGTGATCTG 5' and WRN-2: 5' ATCCTGTGGAACATACCATG 3' inserted by restriction cloning into the pCAG-eCas9-GFP-U6-gRNA (pCAG-eCas9-GFP-U6-gRNA was a gift from Jizhong Zou (Addgene plasmid # 79145; http://n2t.net/addgene:79145 ; RRID:Addgene_79145) at Bbs1 restriction sites. Cells were transfected according to the manufacturer's instructions using lipofectamine 2000 (Thermo Fisher Scientific).

Production of lentiviral particles in HEK-293T cells and transduction of U2OS and HEK-293T cells were performed as previously described (15).

Plasmids and DNA manipulations.

The lentiviral vectors allowing expression of mCherry-tagged human PAXX was described (16).

Ku80 expression vectors: the untagged shRNA-resistant Ku80 and the Ku80-I112R lentiviral expression vector were already described (2).The Ku80-K7E mutant expression vector was obtained by overlap PCR mutagenesis from the untagged shRNA-resistant Ku80 vector using Kpn2-Ku80-K7E-F and pLV-R as mutated inner primers. The PCR fragments were then inserted at the Kpn2I-BcuI restriction site of the pLV3 vector backbone.

Ku70 expression vectors: the Ku70-mutant expression vector was obtained by PCR amplification of the Ku70 coding sequence from the previously described pLV3-FLAG-shR-Ku70 vector (14) using Kpn2-Ku70-F and Bcu-Ku70-R as outer primers and Ku70-mut-F and Ku70-mut-R as inner primers. The PCR fragments were then inserted at the Kpn2I-BcuI restriction site of the pLV3 vector backbone using the Hot-Fusion strategy. The expression vector for mCherry-tagged human Ku70 was constructed by PCR amplification of Ku70 cDNA with Mlu-Ku70-F and Bcu-Ku70-R primers and insertion by Hot-Fusion between MluI and BcuI restriction sites downstream the coding sequence of mCherry previously inserted in the pLV3 vector.

WRN expression vectors: the GFP-WRN-exo-mutant expression vector were obtained by PCR amplification of the WRN exonuclease domain coding sequence form the pLV-hWRN-shRes vector using Kpn2-WRN-F and Mlu-WRN-236-R as outer primers and WRN-mut-F and WRN-mut-R as mutated inner primers. The PCR fragments were then inserted at the Kpn2I-MluI restriction sites downstream the coding sequence of GFP previously inserted in the pLV3 vector.

The pLV-hWRN-shRes vector was obtained by PCR amplification of the human WRN cDNA (kind gift of Dr Karlseder, (17)) using hWRN-Mlu-F and hWRN-Xma-R primers. The resulting PCR fragment was then inserted at the MluI-XmaI restriction site in the previously described pLV-tTR-KRAB vector (2).

Nucleofection

KO WRN U2OS cells (U2OS WRN KO) were transiently nucleofected with pLVX-WRN wild-type, pLVX-WRN R149E and pLVX-WRN E84A plasmids. 10 µg of DNA were used for 1.5 $\times 10^6$ cells, with 2 pulses of 950 V lasting 30 ms by Invitrogen Neon Transfection system (Invitrogen). Afterwards, cells were seeded in 10% FBS medium to carry out the different experimental assays.

Western blotting analysis

Western blotting analysis was performed using standard methods. Blots were incubated with primary antibodies against LAMIN B1 (Abcam AB16048, 1:20000), WRN (Abcam AB200, 1:2000), GAPDH (Millipore mab374, 1:10000), Ku70 (Santa Cruz Biotechnology ab92450, 1:1000).

After incubations with horseradish peroxidase-linked secondary antibodies (Jackson immunosciences), the blots were developed using the chemiluminescence detection kit ECL-Plus (Amersham) according to the manufacturer's instructions.

SUPPLEMENTARY REFERENCES

1. Gorda, B., Toelzer, C., Aulicino, F. and Berger, I. The MultiBac BEVS: Basics, applications, performance and recent developments. *Methods Enzymol*, **660**, 129-154 (2021).
2. Nemoz, C., Ropars, V., Frit, P., Gontier, A., Drevet, P., Yu, J., Guerois, R., Pitois, A., Comte, A., Delteil, C. *et al.* XLF and APLF bind Ku80 at two remote sites to ensure DNA repair by non-homologous end joining. *Nature Structural & Molecular Biology*, **25**, 971-980 (2018).
3. Pinto, C., Kasaciunaite, K., Seidel, R. and Cejka, P. Human DNA2 possesses a cryptic DNA unwinding activity that functionally integrates with BLM or WRN helicases. *eLife*, **5** (2016).
4. Reginato G, Cannavo E, Cejka P. Physiological protein blocks direct the Mre11-Rad50-Xrs2 and Sae2 nuclease complex to initiate DNA end resection. *Genes Dev*. **31(**23-24):2325-2330 (2017).
5. Mengoli V, Ceppi I, Sanchez A, Cannavo E, Halder S, Scaglione S, Gaillard PH, McHugh PJ, Riesen N, Pettazzoni P, Cejka P. WRN helicase and mismatch repair complexes independently and synergistically disrupt cruciform DNA structures. *EMBO J,* **42**(3):e111998. doi: 10.15252/embj.2022111998. Epub 2022 Dec 21 (2023).
6. Walker, J.R., Corpina, R.A. and Goldberg, J. Structure of the Ku heterodimer bound to DNA and its implications for double-strand break repair. *Nature*, **412**, 607-614 (2001).
7. Tegunov D, Cramer P. Real-time cryo-electron microscopy data preprocessing with Warp. *Nat Methods.* **16**(11):1146-1152 (2019).
8. Punjani, A., Rubinstein, J.L., Fleet, D.J. and Brubaker, M.A. cryoSPARC: algorithms for rapid unsupervised cryo-EM structure determination. *Nature methods*, **14**, 290-296 (2017).
9. Pettersen, E.F., Goddard, T.D., Huang, C.C., Couch, G.S., Greenblatt, D.M., Meng, E.C. and Ferrin, T.E. UCSF Chimera—a visualization system for exploratory research and analysis. *Journal of computational chemistry*, **25**, 1605-1612 (2004).
10. Lohkamp, B., Scott, W. and Cowtan, K. Features and development of Coot. *Acta Crystallographica. Section D: Biological Crystallography*, **66** (2010).
11. Afonine, P.V., Poon, B.K., Read, R.J., Sobolev, O.V., Terwilliger, T.C., Urzhumtsev, A. and Adams, P.D. Real-space refinement in PHENIX for cryo-EM and crystallography. *Acta Crystallographica Section D: Structural Biology*, **74**, 531-544 (2018).
12. Pontén, J. and Saksela, E. Two established in vitro cell lines from human mesenchymal tumours. *Int J Cancer*, **2**, 434-447 (1967).
13. Seif-El-Dahan, M., Kefala-Stavridi, A., Frit, P., Hardwick, S.W., Chirgadze, D.Y., Maia De Oliviera, T., Andreani, J., Britton, S., Barboule, N., Bossaert, M. *et al.* PAXX binding to the NHEJ machinery explains functional redundancy with XLF. *Science Advances*, **9**, eadg2834 (2023).
14. Kefala Stavridi, A., Gontier, A., Morin, V., Frit, P., Ropars, V., Barboule, N., Racca, C., Jonchhe, S., Morten, Michael J., Andreani, J. *et al.* Structural and functional basis of inositol hexaphosphate stimulation of NHEJ through stabilization of Ku-XLF interaction. *Nucleic Acids Research*, **51**, 11732-11747 (2023).
15. Cheng, Q., Barboule, N., Frit, P., Gomez, D., Bombarde, O., Couderc, B., Ren, G.-S., Salles, B. and Calsou, P. Ku counteracts mobilization of PARP1 and MRN in chromatin damaged with DNA double-strand breaks. *Nucleic Acids Research*, **39**, 9605-9619 (2011).
16. Frit, P., Amin, H., Zahid, S., Barboule, N., Hall, C., Matharu, G., Hardwick, S.W., Chauvat, J., Britton, S., Chirgadze, D.Y. *et al.* DNA polymerase Lambda is anchored within the NHEJ synaptic complex via Ku70/80. *bioRxiv*, 2024.2008.2012.607588 (2024).
17. Crabbe, L., Verdun, R.E., Haggblom, C.I. and Karlseder, J. Defective telomere lagging strand synthesis in cells lacking WRN helicase activity. *Science*, **306**, 1951-1953 (2004).
