# Supplementary material for: Structural basis of Ku-mediated activation of WRN exonuclease activity
Source: Nat Commun. 2026 May 13;17:6369. doi: 10.1038/s41467-026-71888-w (PMC13377182; doi:10.1038/s41467-026-71888-w)
Supplement: Supplementary file 2 — Reporting Summary [file 41467_2026_71888_MOESM2_ESM.pdf]

nature portfolio

Corresponding author(s): Patrick Calsou and Virginie Ropars

Last updated by author(s): Mar 15, 2026

Reporting Summary

Nature Portfolio wishes to improve the reproducibility of the work that we publish. This form provides structure for consistency and transparency in reporting. For further information on Nature Portfolio policies, see our Editorial Policies and the Editorial Policy Checklist.

Statistics

For all statistical analyses, confirm that the following items are present in the figure legend, table legend, main text, or Methods section.

n/a	Confirmed	
☐	☒	The exact sample size (*n*) for each experimental group/condition, given as a discrete number and unit of measurement
☐	☒	A statement on whether measurements were taken from distinct samples or whether the same sample was measured repeatedly
☐	☒	The statistical test(s) used AND whether they are one- or two-sided<br>*Only common tests should be described solely by name; describe more complex techniques in the Methods section.*
☒	☐	A description of all covariates tested
☐	☒	A description of any assumptions or corrections, such as tests of normality and adjustment for multiple comparisons
☐	☒	A full description of the statistical parameters including central tendency (e.g. means) or other basic estimates (e.g. regression coefficient) AND variation (e.g. standard deviation) or associated estimates of uncertainty (e.g. confidence intervals)
☐	☒	For null hypothesis testing, the test statistic (e.g. *F*, *t*, *r*) with confidence intervals, effect sizes, degrees of freedom and *P* value noted<br>*Give P values as exact values whenever suitable.*
☒	☐	For Bayesian analysis, information on the choice of priors and Markov chain Monte Carlo settings
☐	☒	For hierarchical and complex designs, identification of the appropriate level for tests and full reporting of outcomes
☒	☐	Estimates of effect sizes (e.g. Cohen's *d*, Pearson's *r*), indicating how they were calculated

Our web collection on statistics for biologists contains articles on many of the points above.

Software and code

Policy information about availability of computer code

Data collection	Data from multiphoton laser micro-irradiation were collected on an LSM710 microscope (Zeiss) using the ZEN interface. for Exonuclease assays and EMSA, We used commercial software, available as a package with the respective instrument, for data collection. This includes Typhoon FLA 9500 Phosphor Imager (GE Healthcare, Version 3.0.0.2), Photo scanner operated with Epson Scan v3.9.4.0 US software and CanoScan 9000F Mark II scanner operated with ImageCapture v6.6(525) software. We used the EPU software for the cryo-EM data collection. Immunofluorescent data were acquired using the Image-Pro-Plus 6.0 software (Nikon) or Evident Scientific CellSense. Blots and gels were acquired using the ImageLab software of the ChemiDoc MP apparatus (Bio-Rad) or through ImageJ.
Data analysis	For multiphoton laser micro-irradiation data analysis, use of a custom code on Python 3.12.6. This custom code uses text files containing fluorescence intensity data for irradiated nuclei under a given condition to generate, for each color, a table of intensity ratios, a table of these normalized ratios, and a table combining the mean of the normalized ratios, the standard deviation (SD), and the standard error of the mean (SEM) as a function of time. A second program compiles the conditions to be compared and returns a table for each fluorescence color, grouping together the average of the normalized ratios and the associated standard deviation from the mean for each condition. For Exonuclease assays and EMSA, Data analysis was conducted using only commercially availabe or publicly accessible software. This includes ImageJ2 (NIH, Version 2.9.0/1.53t) for the analysis of gel data; graphs and numerical data (including statistics/error bars) was analyzed and plotted by Prism10 (GraphPad, Version 10.2.3). The cryo-EM data collection was analysed by the Cryosparc v3.0 software. data analyses have been made using the built-in statistical tools of the Prism 9 software from GraphPad Inc.

For manuscripts utilizing custom algorithms or software that are central to the research but not yet described in published literature, software must be made available to editors and reviewers. We strongly encourage code deposition in a community repository (e.g. GitHub). See the Nature Portfolio guidelines for submitting code & software for further information.

Data

Policy information about availability of data

All manuscripts must include a data availability statement. This statement should provide the following information, where applicable:

- Accession codes, unique identifiers, or web links for publicly available datasets
- A description of any restrictions on data availability
- For clinical datasets or third party data, please ensure that the statement adheres to our policy

All relevant data generated or analyzed during this study are included in this article and its supplementary information file. For exonuclease assays and EMSA, Datasets will be uploaded into Mendeley data at the submission of a revised version stage and access will be available if and when the ms will be published. The cryo-EM structure is linked to the PDB code 9HZG and the EMD-52524 map.

Research involving human participants, their data, or biological material

Policy information about studies with human participants or human data. See also policy information about sex, gender (identity/presentation), and sexual orientation and race, ethnicity and racism.

Reporting on sex and gender	n/a
Reporting on race, ethnicity, or other socially relevant groupings	n/a
Population characteristics	n/a
Recruitment	n/a
Ethics oversight	n/a

Note that full information on the approval of the study protocol must also be provided in the manuscript.

Field-specific reporting

Please select the one below that is the best fit for your research. If you are not sure, read the appropriate sections before making your selection.

☒ Life sciences ☐ Behavioural & social sciences ☐ Ecological, evolutionary & environmental sciences

For a reference copy of the document with all sections, see nature.com/documents/nr-reporting-summary-flat.pdf

Life sciences study design

All studies must disclose on these points even when the disclosure is negative.

Sample size	For biphoton laser microirradiation analysis, No sample size calculation was performed a priori. For exonuclease assays and EMSA, Sample size (or number of repeats) was chosen based on what is common in the field, and what was practical to do. For DNA fiber assays, Sample size was not predetermined and was indicated in the figure legends. Sample size was selected on the basis of relevant experience in our lab and previous studies by our and others.
Data exclusions	For multiphoton laser micro-irradiation data analysis, No data were excluded from the analyses. For exonuclease assays and EMSA, In general, no data were excluded unless there was a valid reason to do so, e.g. experiments with failed positive controls indicating technical problems, or when loading control indicated unequal loading that invalidated the analysis or other technical issues (broken gels, collapsed wells in gels etc.). for DNA fiber assays, No data exlcusion was performed.
Replication	Replication was performed and reproducibility of changes was assessed using indicated statistical methods. The experiments were repeated multiple times, as indicated in the figure legend. For DNA fiber assays, All experiments were repeated at least 2 times, unless stated differently in figure legends. The main observations of the work were reproduced in different human cell lines, in different experimental settings and by different technologies.<br>Sample size and number of independent experiments are clearly stated in the figure legend or in methods
Randomization	Randomization is not relevant to the experiments performed in this study. This study is not sensitive to any biased analysis.
Blinding	Blinding was not relevant in our studies, as measurements were objectively quantified by dedicated software or simply visually presented. Furthermore, the loading order or samples on gels prevented blinding. Investigators were not blinded, however, critical experiments were analysed by independent investigators

Reporting for specific materials, systems and methods

We require information from authors about some types of materials, experimental systems and methods used in many studies. Here, indicate whether each material, system or method listed is relevant to your study. If you are not sure if a list item applies to your research, read the appropriate section before selecting a response.

Materials & experimental systems

n/a	Involved in the study
☐	☒ Antibodies
☐	☒ Eukaryotic cell lines
☒	☐ Palaeontology and archaeology
☒	☐ Animals and other organisms
☒	☐ Clinical data
☒	☐ Dual use research of concern
☒	☐ Plants

Methods

n/a	Involved in the study
☒	☐ ChIP-seq
☒	☐ Flow cytometry
☒	☐ MRI-based neuroimaging

Antibodies

Antibodies used

Primary antibodies used:
- mouse monoclonal anti-Ku70 antibody: clone N3H10 from Thermo Fisher Scientific, MA5-13110, lot: WE27669780, (https://www.thermofisher.com/antibody/product/Ku70-Antibody-clone-N3H10-Monoclonal/MA5-13110)
- mouse monoclonal anti-WRN antibody: clone 195C from Sigma-Aldrich, W0393, lot: 037M4751V, (https://www.sigmaaldrich.com/FR/fr/product/sigma/w0393?srsltid=AfmBOopTBQvcvEdJLXbxOIqPAR3cWjEWArifgleb7o9Fgq5eOkXJdu42)
- mouse monoclonal anti-GAPDH antibody: clone GT239 from GenTex, GTX627408, lot: 45770, (https://www.genetex.com/Product/Detail/GAPDH-antibody-GT239/GTX627408?srsltid=AfmBOorYczd69Phw4MKBrM3nQXClUaQIMbA26s5TwDrfsYVR3Tlg1hfe)

Validation

Validations are available from the manufacturers (please refer to the provided link)

Eukaryotic cell lines

Policy information about cell lines and Sex and Gender in Research

Cell line source(s)

U2OS cells (human osteosarcoma cell line) were obtained from the ECACC (Salisbury, UK)
HEK-293T cells (human embryonic cells, CRL-3216) were obtained from the ATCC (Manassas, VA, USA)
Sf21 cells (spodoptera frugiperda) were used from Gibco.

Authentication

The cell lines have not been formally authenticated, except on the basis of morphological criteria and growth rates provided by the supplier

Mycoplasma contamination

Cells were tested negative for mycoplasma and regularly treated with Plasmocure

Commonly misidentified lines
(See ICLAC register)

n/a

Plants

Seed stocks

n/a

Novel plant genotypes

n/a

Authentication

n/a
