# Supplementary material for: Structural basis of Ku-mediated activation of WRN exonuclease activity
Source: Nat Commun. 2026 May 13;17:6369. doi: 10.1038/s41467-026-71888-w (PMC13377182; doi:10.1038/s41467-026-71888-w)
Supplement: Supplementary file 3 — Transparent Peer Review file [file 41467_2026_71888_MOESM3_ESM.pdf]

nature portfolio

Peer Review File

Structural basis of Ku-mediated activation of WRN exonuclease activity

Corresponding Author: Dr Virginie Ropars

This file contains all reviewer reports in order by version, followed by all author rebuttals in order by version.

Version 0:

Reviewer comments:

Reviewer #1

(Remarks to the Author)
The authors present the cryo-EM structure of human Ku70/80 in complex with DNA and the exonuclease domain of WRN. They identify how the elusive SAP domain of Ku70 interacts with the complex and analyze the effects of interface mutations on interaction, UV laser microirradiation and sensitivity to replication stress and crosslink inducing agents. Their experiments support a role for WRN and Ku at replication forks and provide a long awaited role for the Ku70 SAP domain

The manuscript is well written and easy to follow. I have several minor comments and suggestions.

Micrographs should have scale bars

Figure S4C: The authors state that recruitment of Ku to breaks in the I112R mutant was not compromised (bottom of page 8), in fact there seems to be an increase of binding of Ku70-mch in Ku80-I112R , particularly in the first 20 seconds. Is this significant? How do the authors account for this?

Minor point, same figure: I find it visually difficult to distinguish between the black and dark purple lines. Could a different colour be used?

Legend Supplementary S4A: mention blue dot = streptavidin.

Page 11: “some mutations in Ku70 compromised PAXX recruitment”. Please clarify which mutations and how this explains compromised PAXX recruitment.

Throughout, it would help the reader if the region in which the mutation is found could be indicated along with the mutation itself e g page 11: Ku70 K596A (SAP domain)

Most of the experiments have been carried out only in U2OS cells. One panel in Figure 8B was carried out in HAP1 cells. What are HAP1 cells, why was it chosen?

Figures S3D/F and S4B should have statistics. Same for S6F/H

Sex of cell lines is not discussed.

Reviewer #2

(Remarks to the Author)
Zahid et al. manuscript describes a high-resolution cryo-EM structure of the human Ku70/80–DNA complex with WRN, offering insights into the molecular basis of WRN activation during DNA repair and replication stress in the NHEJ pathway. The authors identify key interaction interfaces, between the WRN A-KBMs and the Ku80 vWA domain, and highlight the role of the Ku70 SAP domain in stimulating WRN’s exonuclease activity. Biochemical and cellular assays support the structural

findings by showing that these interactions are critical for efficiently recruiting WRN to damaged DNA.
This study shows the importance of structural interactions between WRN and Ku in facilitating precise DNA end processing and repair. It is of high interest for the community and adds knowledge for future work. Overall, this study is a valuable contribution to the field and, with minor revisions, is recommended for publication.

1- Did the authors consider using full-length WRN for cryo-EM? Understandably, it might be difficult to express alone but did they try to express WRN full length with KU 70/80? If full-length WRN was attempted but unsuccessful, a brief discussion of the challenges encountered would help readers understand the technical limitations.

2- WRN has multiple domains that can influence its interaction with Ku and DNA, especially the helicase and HRDC domains, which could affect the observed conformation or regulatory mechanisms?

3- The manuscript describes the presence of a C-terminal KBM in WRN, but its functional significance isn’t known as described by the authors. Have the authors tested whether this KBM contributes to an alternative binding mode or influences WRN’s exonuclease vs. helicase activities? Could it play a regulatory role distinct from the N-terminal KBM? Or have an additive effect on binding to DNA and Ku70/80 together with N-term KBM?

4- Have the authors considered testing full-length WRN in the presence of DNA-PKcs to determine how phosphorylation might regulate WRN activity and contribute to a more complete working model of the whole DNA-PKcs/Ku70/80/WRN complex? Since DNA-PKcs phosphorylates WRN and regulates its enzymatic activities, testing WRN in a DNA-PKcs/Ku complex would provide deeper insight into its regulation at stressed replication forks.

5- Authors used the DNA substrates that are limited in their structural attributes. WRN is known to work on a diverse set of DNA complex structures like stalled replication fork and G-quadrupoles etc. It would be valuable to test in biochemical assay whether the Ku70/80-WRN interaction similarly regulates exonuclease activity on these physiologically relevant substrates.

6- What features of human Ku enable its enhancement of WRN exonuclease activity on a 5'-overhang substrate, while yeast Ku is inhibitory, and how does this specificity relate to the observed inhibition of WRN activity on blunt-ended DNA?

7- The authors should consider catalytically inactive (dead) WRN mutant during cryo-EM sample preparation to determine whether this could stabilize the missing DNA region and provide better structural resolution, rather than the potential WRN-mediated processing observed in the wild-type structure. This approach would also help clarify their observations regarding WRN’s interaction with the DNA substrate.

8- Given WRN’s potential to adopt different binding modes, did the authors try deep 3D classifications to capture additional functionally relevant states?

9- The authors should make another supplementary figure to show the segments of the density in different regions with electron density fitted with the model.

Reviewer #3

(Remarks to the Author)
In this manuscript entitled "Regulation of WRN exonuclease activity by its interaction with Ku influences the cellular response to replication stress", the authors Zahid, S et al aimed to identify how Ku regulates WRN activity. The authors presented a high resolution cryo-EM structure of the Ku heterodimer and N-terminal exonuclease domain of WRN. The authors identified multiple specific contacts which mediate the WRN and Ku interaction including vWA-like domain in Ku80, the SAP domain in Ku70, and the APLF-like Ku binding domain in WRN. The authors mutated the residues in Ku80, Ku70, and WRN that are responsible for the Ku-WRN interaction to determine if the Ku-WRN interaction modulates WRN exonuclease activity in vitro. The authors clearly show mutating these residues results in a decrease in the Ku-WRN interaction in vitro (using ITC) and in vivo (via localization of WRN to DNA damage induced by 2-photon laser microirradiation). The authors also show that Ku stimulates WRN activity in vitro and that this is lost when the amino acids required for the Ku-WRN interaction are mutated to disrupt the interaction. The data presented in Figures 1-5 are well controlled and of interest to those in the DNA repair field. Typically, I would next list changes/suggestions that I believe would strengthen the paper. However, the end of this manuscript (Figure 6) which drives the title of this interaction "influences the cellular response to replication stress" is significantly lacking that the work does not support the conclusions and claims. For example, the authors characterize (WRN exonuclease activity, recruitment of WRN to DSBs,etc) a number of Ku and WNR mutants in the study but in the response to replication stress, the authors only show that one mutation (Ku70 K596A) results in increased viability to HU exposure compared to WT Ku70. Why did the authors not test their other mutants? Further, as HU exposure results in DNA double strand break (DSB) formation, this increased resistance to HU may simply due to a decreased formation of DSBs and/or the DNA damage response and not necessarily attributed to WRN's role in the cellular response to DNA replication stress. The manuscript is lacking all of the traditional methods for studying response to DNA replication stress such as DNA fiber assays, localization of WRN and Ku to the sites of replication stress, etc. For example, the authors assessed the ability to the Ku mutants to affect WRN's localization to DNA damage induced by microirradiation but this set up induces DSBs and does not necessarily correlate to recruitment to sites of DNA replication stress. The authors provide a model in Figure 6D for how Ku may regulate WRN at stalled replication forks but evidence

provided in the manuscript to support this model is severely lacking. It is due to the lack of evidence of Ku influencing the cellular response to replication stress by regulating WRN exonuclease activity that I cannot support publication of this manuscript at Nature Communications.

Version 1:

Reviewer comments:

Reviewer #1

(Remarks to the Author)
I am satisfied with the revisions made by the authors.

Reviewer #2

(Remarks to the Author)
The authors have addressed my questions, and I am fully satisfied with their revised manuscript. Therefore, I endorse publication.

Reviewer #3

(Remarks to the Author)
The authors have made considerable changes to the manuscript and I now recommend its publication in Nature Communications.

Open Access This Peer Review File is licensed under a Creative Commons Attribution 4.0 International License, which permits use, sharing, adaptation, distribution and reproduction in any medium or format, as long as you give appropriate credit to the original author(s) and the source, provide a link to the Creative Commons license, and indicate if changes were made.
In cases where reviewers are anonymous, credit should be given to 'Anonymous Referee' and the source.
The images or other third party material in this Peer Review File are included in the article’s Creative Commons license, unless indicated otherwise in a credit line to the material. If material is not included in the article’s Creative Commons license and your intended use is not permitted by statutory regulation or exceeds the permitted use, you will need to obtain permission directly from the copyright holder.
To view a copy of this license, visit https://creativecommons.org/licenses/by/4.0/

We would like to thank the reviewers for their time and effort devoted to the evaluation of our work. Their constructive comments and suggestions were very helpful in improving the manuscript.

Following the reviewers' recommendations, we have revised the title and adjusted the presentation of the study to better reflect its main focus. The revised version places greater emphasis on the structural and mechanistic aspects of Ku-mediated regulation of WRN exonuclease activity, while the biochemical and cellular experiments are presented as support and validation of the structural observations. Accordingly, we have revised several sections of the text and figures, added new data (especially new Figures 6 and S8), clarified methodological points, and streamlined the supplementary material.
To adequately address the concerns raised by Reviewer #3, we performed additional experiments in collaboration with a partner specialized in the response to DNA replication stress. These contributions are reflected by the inclusion of three additional co-authors in the revised manuscript.

A detailed, point-by-point response to reviewers comments is provided below.

Reviewer #1 (Remarks to the Author):

The authors present the cryo-EM structure of human Ku70/80 in complex with DNA and the exonuclease domain of WRN. They identify how the elusive SAP domain of Ku70 interacts with the complex and analyze the effects of interface mutations on interaction, UV laser microirradiation and sensitivity to replication stress and crosslink inducing agents. Their experiments support a role for WRN and Ku at replication forks and provide a long awaited role for the Ku70 SAP domain.

The manuscript is well written and easy to follow. I have several minor comments and suggestions.

Micrographs should have scale bars

The omission was inadvertent. We have now added scale bars to micrographs and 2D classes, and the corresponding values are indicated in the figure legends.

Figure S4C: The authors state that recruitment of Ku to breaks in the I112R mutant was not compromised (bottom of page 8), in fact there seems to be an increase of binding of Ku70-mch in Ku80-I112R , particularly in the first 20 seconds. Is this significant? How do the authors account for this?

The I1122R mutation in Ku 80 compromises Ku interaction with protein partners bearing A-KBMs including APLF, WRN and MRI (1). Knowing that Ku sliding and inward accumulation in chromatin is controlled by its association with protein partners like DNA-PKcs (2), we speculate that the loss of these partners may affect Ku binding to break ends like for example by increasing the stoichiometry of Ku molecules per end. Therefore, a non-exclusive explanation for the early increase in Ku mutant binding may reflect an excess of Ku accumulation upon sliding inwards from break ends. Nevertheless, we did not explore this

point further since this control was only intended to exclude a trivial explanation to the decrease of WRN-exo recruitment to laser breaks with the Ku mutant.

Minor point, same figure: I find it visually difficult to distinguish between the black and dark purple lines. Could a different colour be used?

The colours have been changed.

Legend Supplementary S4A: mention blue dot = streptavidin.

We have indicated in the legend of figure S4A that the blue dot corresponds to streptavidin.

Page 11: "some mutations in Ku70 compromised PAXX recruitment". Please clarify which mutations and how this explains compromised PAXX recruitment.

The K596A mutation in Ku70 SAP domain clearly impacts on WRN-exo recruitment while preserving PAXX (and therefore Ku) recruitment to laser DNA damage (**Figure 5C**). In contrast, we could not conclude on the effect of the K596E and Q597A mutations in Ku70 SAP domain on the Ku-WRN interaction since they both impaired PAXX recruitment that is strictly Ku-dependent (**Figure S7B**). Since residues 590-604 in SAP domain are also involved in DNA interaction (3,4), this impairment may reflect a disruption of the interaction between Ku and DNA, that is otherwise preserved with the K596A mutant.

Throughout, it would help the reader if the region in which the mutation is found could be indicated along with the mutation itself e g page 11: Ku70 K596A (SAP domain)

We have clarified the description of the mutated region in the revised manuscript.

Most of the experiments have been carried out only in U2OS cells. One panel in Figure 8B was carried out in HAP1 cells. What are HAP1 cells, why was it chosen?

HAP1 is a human pseudo-haploid cell line highly valuable for gene engineering like gene knock-out (here WRN KO). Following the comments of reviewer #3, we have removed analysis of cell survival from the revised manuscript.

Figures S3D/F and S4B should have statistics. Same for S6F/H.

We have replaced the figures by new graphs including statistics.

Sex of cell lines is not discussed.

The origin of U2OS cells from a female osteosarcoma patient (5) is now referenced in supplemental material of the manuscript.

Reviewer #2 (Remarks to the Author):

Zahid et al. manuscript describes a high-resolution cryo-EM structure of the human Ku70/80–DNA complex with WRN, offering insights into the molecular basis of WRN

activation during DNA repair and replication stress in the NHEJ pathway. The authors identify key interaction interfaces, between the WRN A-KBMs and the Ku80 vWA domain, and highlight the role of the Ku70 SAP domain in stimulating WRN's exonuclease activity. Biochemical and cellular assays support the structural findings by showing that these interactions are critical for efficiently recruiting WRN to damaged DNA. This study shows the importance of structural interactions between WRN and Ku in facilitating precise DNA end processing and repair. It is of high interest for the community and adds knowledge for future work. Overall, this study is a valuable contribution to the field and, with minor revisions, is recommended for publication.

1- Did the authors consider using full-length WRN for cryo-EM? Understandably, it might be difficult to express alone but did they try to express WRN full length with KU 70/80? If full-length WRN was attempted but unsuccessful, a brief discussion of the challenges encountered would help readers understand the technical limitations.

2- WRN has multiple domains that can influence its interaction with Ku and DNA, especially the helicase and HRDC domains, which could affect the observed conformation or regulatory mechanisms?

Response to 1/ and 2/:
We did purify full-length WRN. However, because WRN contains two Ku-binding motifs and our data indicate that the N-terminal motif is more important than the C-terminal (see point 3 below), we focused our cryo-EM studies on this region. We also initiated the structural characterization of full-length WRN in complex with Ku or DNA-PK, but until now we have not been able to obtain a stable associated complex. We plan to explore shorter WRN constructs focusing on the C-terminal region in the presence of Ku, which will be the subject of a future project. We agree that other domains of WRN, such as the helicase and HRDC domains, may influence the conformation and underlying mechanisms, and a comprehensive structural investigation of full-length WRN or the second Ku-binding site in complex with Ku thus presents additional challenges, and would kindly ask the reviewer to consider these experiments above the scope of the current. A note on the experimental limitation was added to the text.

3- The manuscript describes the presence of a C-terminal KBM in WRN, but its functional significance isn't known as described by the authors. Have the authors tested whether this KBM contributes to an alternative binding mode or influences WRN's exonuclease vs. helicase activities? Could it play a regulatory role distinct from the N-terminal KBM? Or have an additive effect on binding to DNA and Ku70/80 together with N-term KBM?

We have tested the individual WRN KBMs mutants (W18G and W1410G) on the 5' overhanged substrate with Ku, and the data show that the N-terminal KBM is clearly more important than the C-terminal KBM (**Figure R1**). Mutation of the C-terminal KBM had no negative impact on WRN stimulation by Ku when the N-terminal KBM was active. However, the C-terminal KBM could partially substitute when the N-KBM was missing. Our structural data suggest that interaction of WRN via the C-KBM will not result in an architecture of the complex compatible with WRN exonuclease going towards the Ku-bound end, so we suggest that the interaction of Ku with C-KBM of WRN may help recruit WRN to the DNA substrate, but WRN will need to reposition itself for the exonucleolytic degradation going towards the end. We hypothesize that the C-terminal KBM may position WRN in a configuration suitable

for its helicase activity to assist DNA2 in long-range resection, but we believe this is a separate topic that is above the scope of the current manuscript.

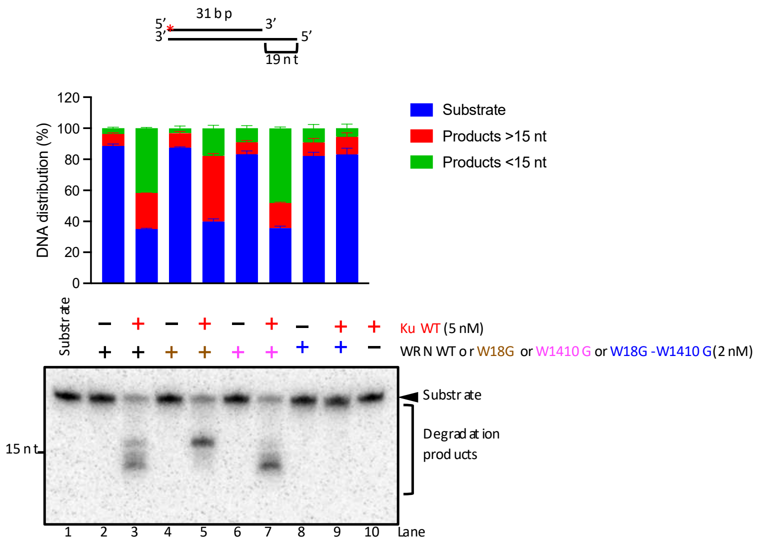

Figure R1. Top, schematic of the substrate. Red asterisk indicates the position of the radioactive label. Middle, quantification of DNA distribution. Average shown; n=3 independent experiments; error bars, SEM. Bottom, representative 15% denaturing polyacrylamide gel showing the degradation of a 5′ overhang (19 nt/31 bp) DNA by the indicated WRN variants in the absence or presence of Ku.

4- Have the authors considered testing full-length WRN in the presence of DNA-PKcs to determine how phosphorylation might regulate WRN activity and contribute to a more complete working model of the whole DNA-PKcs/Ku70/80/WRN complex? Since DNA-PKcs phosphorylates WRN and regulates its enzymatic activities, testing WRN in a DNA-PKcs/Ku complex would provide deeper insight into its regulation at stressed replication forks.

We have only recently prepared DNA-PKcs, and our preliminary data suggest that Ku-DNA-PKcs does not affect the exonuclease activity in the minimal system. Also, we believe that the fraction of WRN that would be phosphorylated by DNA-PKcs could be very small. We have tested pan-WRN phosphorylation (WRN prepared with okadaic acid to preserve phosphorylated sites, labeled as pWRN, prepared in the same way as developed for phosphorylated CtIP) (6) and we saw that with a 5′ overhanged substrate pan-WRN phosphorylation does not play a detectable role in the reaction (**Figure R2**).

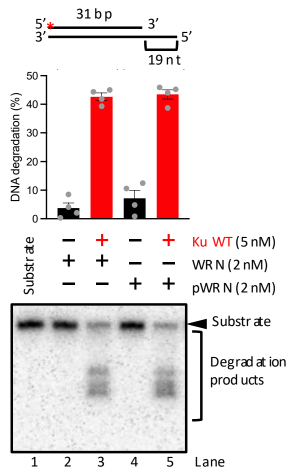

Figure R2. Top, schematic of the substrate. Red asterisk indicates the position of the radioactive label. Middle, quantification of DNA degradation. Average shown; n=4 independent experiments; error bars, SEM. Bottom, representative 15% denaturing polyacrylamide gel showing the degradation of a 5′ overhang (19 nt/31 bp) DNA by regular WRN (labeled as WRN) or pan-WRN phosphorylation (WRN prepared with okadaic acid to preserve phosphorylated sites, labeled as pWRN, prepared in the same way as developed for phosphorylated CtIP) in the absence or presence of Ku.

5- Authors used the DNA substrates that are limited in their structural attributes. WRN is known to work on a diverse set of DNA complex structures like stalled replication fork and G-quadrupoles etc. It would be valuable to test in biochemical assay whether the Ku70/80-WRN interaction similarly regulates exonuclease activity on these physiologically relevant substrates.

Our data (see below) and previous literature suggest that WRN exonuclease needs to start from an overhang (free end), on a substrate that is bound by Ku on the other side, at a certain distance from each other. Hence, the need to position Ku at a free DNA end that contacts WRN limits the possibility and relevance of experiments where secondary structures are introduced. We believe Ku will not regulate WRN if there are secondary DNA structures in between that can block the interaction.

6- What features of human Ku enable its enhancement of WRN exonuclease activity on a 5'-overhang substrate, while yeast Ku is inhibitory, and how does this specificity relate to the observed inhibition of WRN activity on blunt-ended DNA?

We have varied the length of the duplex DNA between the Ku-bound blunt end and the overhang from where WRN initiates exonucleolytic DNA degradation. In case of 31 bp-long duplex (substrate used in a majority of experiments shown in this manuscript), the stimulation is substantial (about 2.4 fold, see left gel in new **Figure S4A**). When we increased the length of the duplex to 50 bp, i.e. above the combined footprints of Ku and WRN, the stimulation decreased to 1.4 fold (see right gel in new **Figure S4A**). When we blocked the blunt end with streptavidin which prevented Ku binding, the stimulation was completely abolished (see middle gel in new **Figure S4A**). On DNA duplexes with blunt ends on both sides, the stimulation was also not apparent, most likely because both ends are bound by Ku, and Ku outcompetes WRN before they can form a functional complex (**Figure S3F**). Therefore, the stimulation is apparent (a) when Ku and WRN bind the same DNA

molecule (one side blunt-ended, the other side overhanged), up to a certain distance from each other, mostly because the stimulation involves interaction dependent on a KBM-mediated physical complex, which is not possible with yeast Ku. These data together support our model that Ku-WRN interaction and resulting activation of WRN exonuclease activity helps displace Ku from DNA ends at reversed forks (see Discussion).

7- The authors should consider catalytically inactive (dead) WRN mutant during cryo-EM sample preparation to determine whether this could stabilize the missing DNA region and provide better structural resolution, rather than the potential WRN-mediated processing observed in the wild-type structure. This approach would also help clarify their observations regarding WRN's interaction with the DNA substrate.

We purified the exonuclease-dead WRN mutant (WRN-exo E84A). However, despite comparable affinity for Ku, we were not able to obtain a stable Ku–DNA–WRNexoE84A complex in cryo-EM grids. Under these conditions, only the Ku–DNA complex could be visualized, preventing further structural characterization of the mutant in this context.

8- Given WRN's potential to adopt different binding modes, did the authors try deep 3D classifications to capture additional functionally relevant states?

We did indeed attempt to separate minor states/subtle conformational variations in the structure - specifically by utilising the 3D classification tool within Cryosparc (with an emphasis on using a focus mask around the Wrn-Exo part of the structure). However, in our hands this did not reveal any additional structural states or improve overall map quality, so use of 3D classification was omitted from our data-processing workflow.

9- The authors should make another supplementary figure to show the segments of the density in different regions with electron density fitted with the model.

Following this advice, we have now included **Figure S5E**, which shows segments of the cryo-EM density fitted with the model in different regions.

Reviewer #3 (Remarks to the Author):

In this manuscript entitled "Regulation of WRN exonuclease activity by its interaction with Ku influences the cellular response to replication stress", the authors Zahid, S et al aimed to identify how Ku regulates WRN activity. The authors presented a high resolution cryo-EM structure of the Ku heterodimer and N-terminal exonuclease domain of WRN. The authors identified multiple specific contacts which mediate the WRN and Ku interaction including vWA-like domain in Ku80, the SAP domain in Ku70, and the APLF-like Ku binding domain in WRN. The authors mutated the residues in Ku80, Ku70, and WRN that are responsible for the Ku-WRN interaction to determine if the Ku-WRN interaction modulates WRN exonuclease activity in vitro. The authors clearly show mutating these residues results in a decrease in the Ku-WRN interaction in vitro (using ITC) and in vivo (via localization of WRN to DNA damage induced by 2-photon laser microirradiation). The authors also show that Ku stimulates WRN activity in vitro and that this is lost when the amino acids required for the Ku-WRN interaction are mutated to disrupt the interaction. The data presented in Figures 1-5 are well controlled and of interest to those in the DNA repair field.

Typically, I would next list changes/suggestions that I believe would strengthen the paper. However, the end of this manuscript (Figure 6) which drives the title of this interaction "influences the cellular response to replication stress" is significantly lacking that the work does not support the conclusions and claims.

For example, the authors characterize (WRN exonuclease activity, recruitment of WRN to DSBs,etc) a number of Ku and WNR mutants in the study but in the response to replication stress, the authors only show that one mutation (Ku70 K596A) results in increased viability to HU exposure compared to WT Ku70. Why did the authors not test their other mutants? Further, as HU exposure results in DNA double strand break (DSB) formation, this increased resistance to HU may simply due to a decreased formation of DSBs and/or the DNA damage response and not necessarily attributed to WRN's role in the cellular response to DNA replication stress.

We thank the reviewer for raising these points. The reviewer is correct that the title reflected data that were presented only towards the end of the manuscript, and that the title was therefore not representative of the whole study. We have changed the title (Structural basis of Ku-mediated activation of WRN exonuclease activity) and text to clarify where the focus and main weight of the study is: determination of structure of Ku interacting with segments of WRN, and the unexpected observation that WRN binds on the internal side of Ku. The following biochemical and cellular data were carried out to validate the structure and the observed interactions and to bring functional significance of these interactions. We would therefore like to ask the reviewer not to consider the manuscript as a "DNA replication stress" paper, as the whole repertoire of functional assays would be above the scope of the current structure-function story.

The manuscript is lacking all of the traditional methods for studying response to DNA replication stress such as DNA fiber assays, localization of WRN and Ku to the sites of replication stress, etc.

To handle the reviewer's concern, we developed a collaboration with Pietro Pichierri. Seminal work from Pichierri lab described that the exonuclease activity of WRN is required to suppress pathological nascent DNA degradation after fork reversal at low-dose of camptothecin (CPT) as model treatment (7). Under these conditions, several dedicated experiments were then performed to test the effect of impaired interaction of Ku70 SAP with WRN on its recruitment and function at perturbed replication forks (new **Figure 6** and **Supplementary Figure 8)**. The impact of disrupted WRN-Ku70 interaction on the localization of WRN at the perturbed replication forks was tested by EdU-PLA (SIRF assay) (**Figure 6A**) and more specifically at the exposed nascent strand of reversed forks by native IdU-PLA assays (**Figure S8C**). Moreover, the fork degradation phenotype (**Figure 6B**) and the fork recovery of cells expressing the interaction-defective allele of WRN R149E were analyzed (**Figure 6C**).
We now show that expression of the WRN mutant R149E or expression of K596A Ku70 do not impair recruitment of full-length WRN to stalled replication forks or to exposed nascent ssDNA formed at the regressed arm of the reversed forks. However, loss of Ku70-WRN interaction phenocopies the loss of WRN exonuclease activity for what concerns the fork degradation phenotype of CPT-treated cells. Nevertheless, this fork degradation does not

seem sufficient to significantly affect the ability of WRN R149E cells to restart CPT-perturbed replication forks. This latter result is not at odds with the degradation-prone phenotype of these cells. Indeed, since the seminal work of Jasin's lab in BRCA2-deficient cells (8), it is widely reported that fork degradation phenotype is uncoupled from the ability to restart replication once the drug that induces fork stalling is washed out. Fork degradation in the absence of BRCA2 engages a recombination-based mechanism for fork recovery (9), and it is possible that the same happens when the exonuclease activity of WRN is affected by either an inactivating mutation (E84A) or a defective in Ku70 binding. Indeed, loss of WRNexo stimulates RAD51 foci formation (10) suggesting that HR is upregulated.

These new results clearly demonstrate that localization of WRN at stalled replication forks is not dependent on the association with Ku but that WRN protective function, dependent on the exonuclease activity, relies on its binding to Ku.

Finally, we acknowledge the limitation of some experiments presented in our initial study. Whatever cell viability test is used, long-term treatment with HU or CPT can result in secondary DSBs, which can reflect a requirement of WRN-exo in DSBs repair, as the reviewer correctly pointed out. Therefore, we have removed this dataset from the revised manuscript.

For example, the authors assessed the ability to the Ku mutants to affect WRN's localization to DNA damage induced by microirradiation but this set up induces DSBs and does not necessarily correlate to recruitment to sites of DNA replication stress.

We and others have previously used protein recruitment to laser breaks to characterize the effect of mutations in either partner on their interaction (11-14). Here, since we found that WRN-exo recruitment to laser breaks was strictly Ku-dependent, it was used as a readout of the impact of mutations deduced from the structure on the Ku-WRN interaction. We agree that it does not correlate to recruitment to sites of DNA replication stress, and the respective manuscript text was modified to acknowledge this. We also acknowledge that our data do not exclude that WRN-Ku interaction plays a role in DSB repair.

The authors provide a model in Figure 6D for how Ku may regulate WRN at stalled replication forks but evidence provided in the manuscript to support this model is severely lacking. It is due to the lack of evidence of Ku influencing the cellular response to replication stress by regulating WRN exonuclease activity that I cannot support publication of this manuscript at Nature Communications.

We agree that our previous model was too speculative and may need further experiments beyond the focus of the present work. Therefore, we changed the title of the manuscript, we simplified the schema in new **Figure 6D** and we reduced the extent of speculation in the discussion section.

References

1. Grundy, G.J., Rulten, S.L., Arribas-Bosacoma, R., Davidson, K., Kozik, Z., Oliver, A.W., Pearl, L.H. and Caldecott, K.W. (2016) The Ku-binding motif is a conserved module for recruitment and stimulation of non-homologous end-joining proteins. *Nature Communications*, **7**, 11242.
2. Bossaert, M., Moreno, A.T., Peixoto, A., Pillaire, M.J., Chanut, P., Frit, P., Calsou, P., Loparo, J.J. and Britton, S. (2024) Identification of the main barriers to Ku accumulation in chromatin. *Cell Rep*, **43**, 114538.
3. Zhang, Z., Zhu, L., Lin, D., Chen, F., Chen, D.J. and Chen, Y. (2001) The three-dimensional structure of the C-terminal DNA-binding domain of human Ku70. *The Journal of biological chemistry*, **276**, 38231-38236.
4. Hall, C., Frit, P., Kefala-Stavridi, A., Pelletier, A., Hardwick, S.W., Amin, H., Bilyard, M.K., Maia De Oliviera, T., Tariq, A., Zahid, S. *et al.* (2025) Cryo-EM structures of NHEJ assemblies with nucleosomes. *Nat Commun*, **17**, 648.
5. Pontén, J. and Saksela, E. (1967) Two established in vitro cell lines from human mesenchymal tumours. *Int J Cancer*, **2**, 434-447.
6. Anand, R., Ranjha, L., Cannavo, E. and Cejka, P. (2016) Phosphorylated CtIP Functions as a Co-factor of the MRE11-RAD50-NBS1 Endonuclease in DNA End Resection. *Mol Cell*, **64**, 940-950.
7. Iannascoli, C., Palermo, V., Murfuni, I., Franchitto, A. and Pichierri, P. (2015) The WRN exonuclease domain protects nascent strands from pathological MRE11/EXO1-dependent degradation. *Nucleic Acids Research*, **43**, 9788-9803.
8. Schlacher, K., Christ, N., Siaud, N., Egashira, A., Wu, H. and Jasin, M. (2011) Double-strand break repair-independent role for BRCA2 in blocking stalled replication fork degradation by MRE11. *Cell*, **145**, 529-542.
9. Lemaçon, D., Jackson, J., Quinet, A., Brickner, J.R., Li, S., Yazinski, S., You, Z., Ira, G., Zou, L., Mosammaparast, N. *et al.* (2017) MRE11 and EXO1 nucleases degrade reversed forks and elicit MUS81-dependent fork rescue in BRCA2-deficient cells. *Nat Commun*, **8**, 860.
10. Aiello, F.A., Palma, A., Malacaria, E., Zheng, L., Campbell, J.L., Shen, B., Franchitto, A. and Pichierri, P. (2019) RAD51 and mitotic function of mus81 are essential for recovery from low-dose of camptothecin in the absence of the WRN exonuclease. *Nucleic Acids Res*, **47**, 6796-6810.
11. Frit, P., Amin, H., Zahid, S., Barboule, N., Hall, C., Matharu, G., Hardwick, S.W., Chauvat, J., Britton, S., Chirgadze, D.Y. *et al.* (2025) Structural and functional insights into the interaction between Ku70/80 and Pol X family polymerases in NHEJ. *Nat Commun*, **16**, 4208.
12. Kefala Stavridi, A., Gontier, A., Morin, V., Frit, P., Ropars, V., Barboule, N., Racca, C., Jonchhe, S., Morten, Michael J., Andreani, J. *et al.* (2023) Structural and functional basis of inositol hexaphosphate stimulation of NHEJ through stabilization of Ku-XLF interaction. *Nucleic Acids Research*, **51**, 11732-11747.
13. Nemoz, C., Ropars, V., Frit, P., Gontier, A., Drevet, P., Yu, J., Guerois, R., Pitois, A., Comte, A., Delteil, C. *et al.* (2018) XLF and APLF bind Ku80 at two remote sites to ensure DNA repair by non-homologous end joining. *Nature Structural & Molecular Biology*, **25**, 971-980.

14. Seif-El-Dahan, M., Kefala-Stavridi, A., Frit, P., Hardwick, S.W., Chirgadze, D.Y., Maia De Oliviera, T., Andreani, J., Britton, S., Barboule, N., Bossaert, M. *et al.* (2023) PAXX binding to the NHEJ machinery explains functional redundancy with XLF. *Science Advances*, **9**, eadg2834.

Below we respond point by point to the referees' comments.
Reviewer #1
We thank the reviewer for their positive assessment and are pleased that they are satisfied with the revisions made.

Reviewer #2
We thank the reviewer for their positive evaluation and for endorsing publication of the revised manuscript.

Reviewer #3
We thank the reviewer for their positive comments and recommendation for publication in Nature Communications.
